# Catalytic interfacial construction of a Li–Al–F-rich SEI for robust silicon dendrite anodes

**DOI:** 10.1039/d6sc04327e

**Published:** 2026-07-20

**Authors:** Xiang Wang, Xiaofan Liu, Yinjiang Du, Yue Lu, Xinyue Dong, Wenqing Ma, Xiangping Chen, Jiang Yin, Yahui Yang, Yanqing Lai, Xiongwei Wu, Lishan Yang

**Affiliations:** a College of Chemistry and Chemical Engineering, Hunan Normal University Changsha 410081 China lsyang@hunnu.edu.cn; b Institute for Advanced Interdisciplinary Research (iAIR), Collaborative Innovation Center of Technology and Equipment for Biological Diagnosis and Therapy in Universities of Shandong, Shandong Key Laboratory of Functional Materials for Integrated Lithium Niobate Photonics, Core Research Facilities, School of Chemistry and Chemical Engineering, University of Jinan Jinan 250022 China ifc_mawq@ujn.edu.cn; c School of Metallurgy and Environment, Central South University Changsha 410083 China

## Abstract

The unstable solid–electrolyte interphase (SEI) on silicon anodes remains a bottleneck for sustainable lithium storage. Silicon dendrites (SD) derived from Al–Si alloys have been widely investigated, yet the residual Al from dealloying is typically overlooked or regarded as an inert impurity. Here, we introduce a defect-to-design strategy that repurposes this residual Al into an interfacial catalyst. By controlling HF etching kinetics, the native thick Li^+^-blocking SiO_*x*_ layer is tailored to an ultrathin thickness, while residual Al is *in situ* converted into uniformly distributed nanometric AlF_3_ on the SD (SD@AlF_3_). This AlF_3_ precursor dynamically evolves into a Li–Al–F-rich interphase, which preferentially adsorbs FEC and PF_6_^−^ and lowers their dissociation barriers, catalyzing a mechanically robust, inorganic-dominated SEI (Young's modulus of 12.0 GPa). Consequently, the optimized SD@AlF_3_ anode exhibits ∼80% suppression in electrode swelling and 81.9% capacity retention after 200 cycles (in a half-cell configuration). Moreover, the SD@AlF_3_‖LFP full cell retains 92.1% of its capacity after 100 cycles at 0.5C. This work highlights intrinsic defect repurposing as an effective route for interfacial regulation in high-capacity alloy anodes.

## Abstract 2

Residual Al in silicon dendrites is repurposed into AlF_3_ nanocatalysts, constructing a Li–Al–F-rich SEI that suppresses electrode swelling and enables long-term stable cycling.

## Graphical abstract

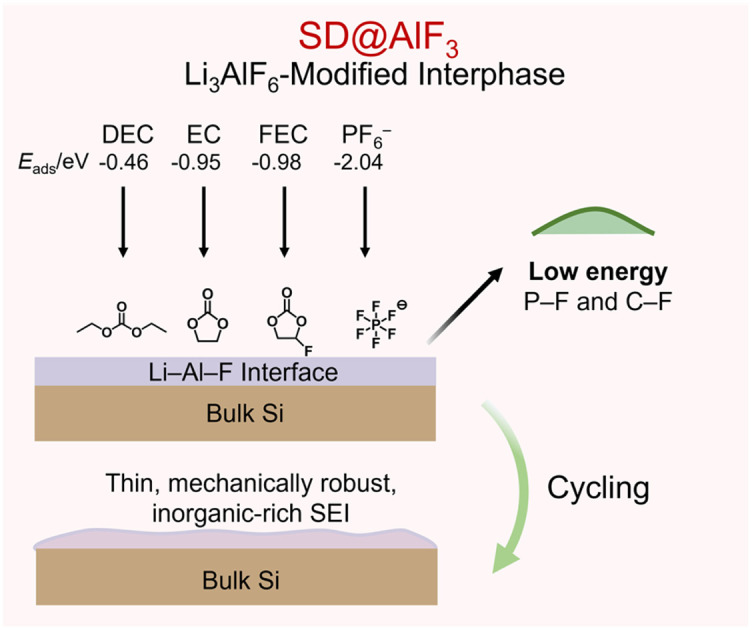

## 1. Introduction

Silicon (Si) stands as the preeminent candidate for next-generation lithium-ion batteries owing to its high theoretical capacity (3579 mAh g^−1^ based on the Li_15_Si_4_ phase) and low operating potential.^1–3^ However, its practical deployment is constrained by rapid capacity decay stemming from drastic volume changes (>300%) during (de)lithiation.^4–6^ These fluctuations fracture the solid-electrolyte interphase (SEI), leading to lithium inventory depletion and structural instability.^7–9^ Recent studies emphasize that the integrity and properties of the SEI, rather than the bulk Si material alone, are paramount in dictating the cycling stability and durability of Si anodes.^10–14^ Thus, proactively engineering a stable and functional SEI is paramount for unlocking the practical potential of Si.

Among various silicon architectures designed to mitigate volume stress, silicon dendrites (SD) derived from dealloying of aluminum (Al)–Si alloys have attracted substantial attention owing to their interconnected dendritic framework with intrinsic void space, which accommodates expansion and offers a scalable synthesis route.^15–18^ In parallel, surface SiO_*x*_ layers generated during dealloying or post-etching treatments have been shown to dictate the interfacial chemistry by modulating SEI composition, ion transport, and mechanical compliance.^19–22^ However, a pervasive yet overlooked feature of this dealloying system is the inevitable presence of residual Al, originating from diffusion-limited dealloying and encapsulation within the silicon matrix.^18,23,24^ Due to its low content (∼3 wt%) and scattered distribution, residual Al is commonly presumed to be fully removed or, when detected, regarded as an inert impurity,^15,18,25–28^ with discussions largely limited to minor electronic or structural effects rather than interfacial chemical reactivity.^23,24^ This perspective represents a significant knowledge gap, as the potential chemical reactivity of these atomic-scale “defects” at the critical electrode–electrolyte interface remains entirely unexplored. In particular, their potential synergy with surface-modifying etching processes to actively regulate interfacial evolution has not been considered.

**Fig. 1:**
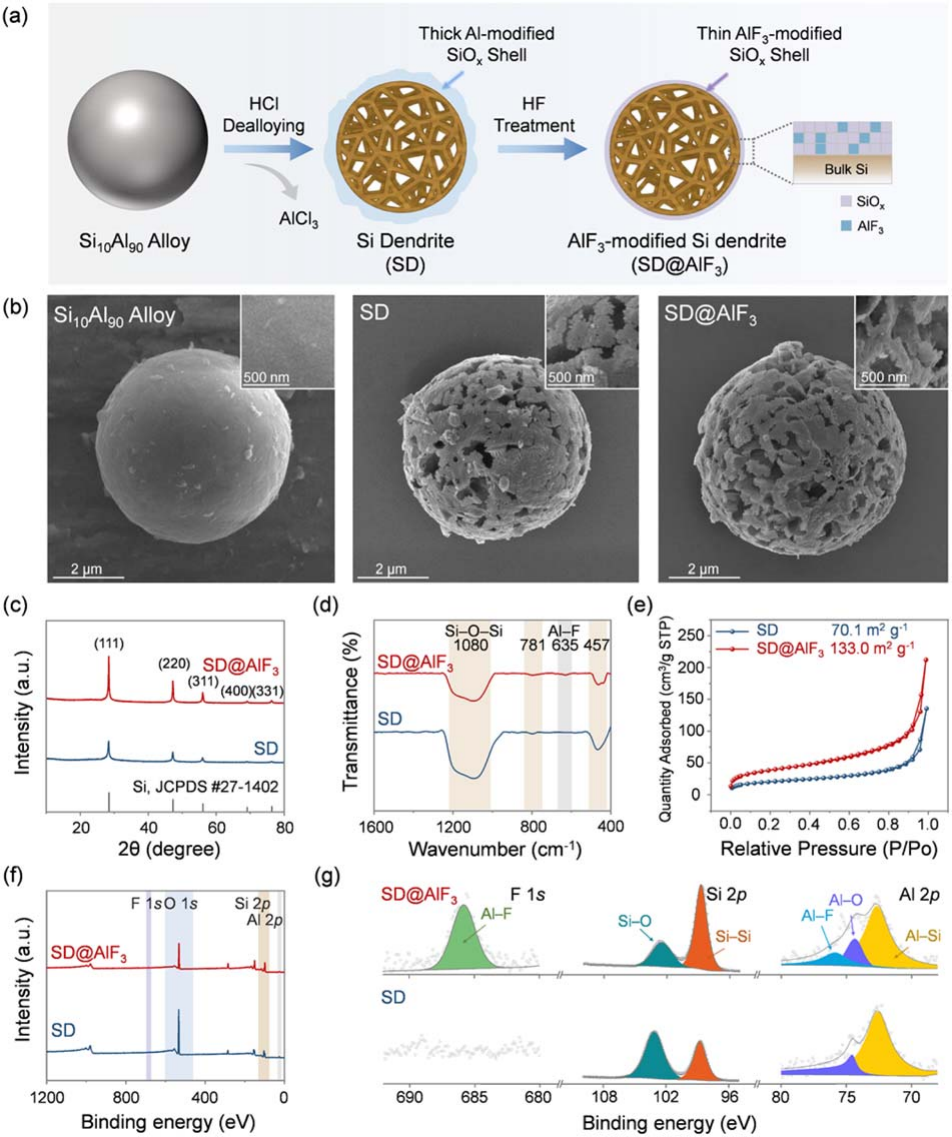
Surface chemical and compositional evolution. (a) Schematic of the “defect-to-design” strategy from the Si_10_Al_90_ alloy to SD@AlF_3_. (b) SEM images of Si_10_Al_90_ alloy, SD, and SD@AlF_3_. (c) XRD patterns, (d) FTIR spectra, (e) N_2_ adsorption–desorption isotherms, (f) XPS survey, and (g) high-resolution Si 2p, Al 2p, and F 1s spectra of SD and SD@AlF_3_.

Herein, we report a defect-to-design interfacial engineering strategy that transforms overlooked residual Al in dealloyed porous silicon into an active regulator of SEI formation. By precisely regulating HF etching kinetics, we simultaneously optimize the surface SiO_*x*_ layer to an ultrathin thickness of ∼2 nm and trigger the *in situ* conversion of residual Al into uniformly distributed nanometric AlF_3_ on the SD (SD@AlF_3_) (Fig. 1a). During electrochemical activation, this AlF_3_ precursor evolves into a Li–Al–F-rich interphase. Crucially, this interphase preferentially adsorbs FEC and PF_6_^−^ and catalytically lowers their dissociation barriers, thereby guiding the formation of a thin, inorganic-dominated, and mechanically robust SEI. This interphase exhibits high mechanical robustness and electronic passivation, as evidenced by a Young's modulus of 12.0 GPa and a uniform surface potential of 0.55 V. As a consequence, electrode swelling is suppressed by ∼80% compared to that of SD, enabling markedly improved cycling stability. This work not only uncovers the previously neglected chemical role of residual Al in Al–Si dealloying systems but also establishes a scalable paradigm for transforming intrinsic defects into functional interfacial regulators for high-capacity alloy anodes.

## 2. Experimental

### 2.1. Materials preparation

The SD were synthesized by chemical dealloying of commercial Si_10_Al_90_ alloy powder (average particle size ∼ 5 µm, Changsha Tianjiu Metal Materials Co., Ltd). Typically, 10 g of alloy powder was dispersed in 200 mL of 3 M hydrochloric acid (HCl) solution and stirred at 300 rpm for 1.5 h at room temperature. The acid leaching process was repeated three times to thoroughly remove the aluminum matrix. The resulting powder was washed with deionized water until the supernatant reached neutral pH. To fabricate the AlF_3_-modified samples, the acid-leached silicon was immersed in 100 mL of hydrofluoric acid (HF) solution with controlled concentrations (2, 5, and 8 wt%) for 5 min at room temperature. The reaction was quenched by diluting with a large excess of deionized water, followed by repeated washing until neutral. The final products were vacuum-dried at 80 °C for 6 h to obtain micro-sized dendritic silicon powders, denoted as SD@AlF_3_-2, SD@AlF_3_, and SD@AlF_3_-8, corresponding to the HF concentrations used.

### 2.2. Electrode fabrication and cell assembly

An aqueous sodium alginate (Alg) binder solution was prepared by dissolving Alg powder in deionized water at a concentration of 0.02 g mL^−1^ under continuous stirring at room temperature for 3–4 h until a homogeneous solution was obtained. Working electrodes were fabricated by a slurry-casting method. The active material, Super P conductive carbon, and Alg binder were mixed in a weight ratio of 70 : 15 : 15. Deionized water was added to form a homogeneous slurry, which was then uniformly cast onto a copper foil current collector using a doctor blade with a 100 µm gap. The coated electrode was air-dried at room temperature, followed by vacuum drying at 100 °C for 12 h. The dried electrodes were punched into discs and stored in an argon-filled glovebox. The areal mass loading of the active material was approximately 0.50 mg cm^−2^.

CR2032 coin-type half-cells were assembled in an argon-filled glovebox (O_2_ and H_2_O levels < 0.1 ppm). The prepared silicon electrode was used as the working electrode, metallic lithium foil as the counter/reference electrode, and a Celgard 2025 membrane as the separator. The electrolyte was 1 M LiPF_6_ in a mixture of ethylene carbonate (EC) and diethyl carbonate (DEC) (1 : 1 by volume) with 10 wt% fluoroethylene carbonate (FEC) as an additive. The cells were sealed under 50 MPa pressure and aged at 30 °C for 24 h before electrochemical testing.

### 2.3. Materials characterization

X-ray diffraction (XRD) patterns were collected on a Bruker D8 Advance diffractometer with Cu Kα radiation (*λ* = 1.5406 Å) over a 2*θ* range of 10°–80°. Fourier-transform infrared (FTIR) spectroscopy was performed on a Thermo Scientific Nicolet iS20 spectrometer in the range of 400–4000 cm^−1^. X-ray photoelectron spectroscopy (XPS) analysis was conducted using a Thermo Scientific K-Alpha spectrometer. Morphology and elemental mapping were examined by field-emission scanning electron microscopy (FESEM, Zeiss Sigma 300) and transmission electron microscopy (TEM, JEOL JEM-F200). N_2_ adsorption–desorption isotherms were measured at 77 K using a Micromeritics ASAP 2460 analyzer. The specific surface area was calculated by the Brunauer–Emmett–Teller (BET) method, and pore size distribution was derived using the Barrett–Joyner–Halenda (BJH) model. Contact angles were measured using a KINO TX500™ spinning drop tensiometer with carbonate-based electrolyte (1 M LiPF_6_ in EC/DEC (1 : 1 by volume) with 10 wt% FEC additive) as the probe liquid. Atomic force microscopy (AFM) was performed on a Bruker Dimension Icon system in PeakForce QNM and Kelvin probe force microscopy (KPFM) modes with a scan area of 2 × 2 µm^2^. Time-of-flight secondary ion mass spectrometry (TOF-SIMS) depth profiling was conducted on a TOF.SIMS 5-100 instrument (IONTOF GmbH) using a 30 keV Bi^+^ primary ion beam for analysis and a 1 keV Cs^+^ beam for sputtering (sputter area: 200 × 200 µm^2^).

### 2.4. Electrochemical measurements

Galvanostatic charge/discharge (GCD) tests were performed on a Neware CT-4008 battery test system within a voltage window of 0.01–2.00 V (*vs.* Li^+^/Li) at 30 °C. The cells underwent a conditioning step at 0.2 A g^−1^ for the first 3 cycles, followed by 3 additional conditioning cycles at 1 A g^−1^. Long-term cycling stability was then evaluated at 1 A g^−1^, with the capacity retention rate calculated from the 6th cycle onward. Rate capability was evaluated by applying stepwise current densities of 0.2, 0.5, 1, 2, and 3 A g^−1^, with each rate sustained for 5 cycles, before returning to 0.2 A g^−1^ for a final 5 cycles to assess capacity recovery. Cyclic voltammetry (CV) was conducted on a CHI760E electrochemical workstation at scan rates from 0.1 to 0.5 mV s^−1^. Electrochemical impedance spectroscopy (EIS) measurements were carried out on the same workstation with an AC amplitude of 5 mV over a frequency range of 100k to 0.01 Hz. Galvanostatic intermittent titration technique (GITT) tests were performed using the Neware system by applying a constant current pulse of 0.2 A g^−1^ for 10 min, followed by an open-circuit relaxation for 1 h. Full-cell tests were conducted using commercial LiFePO_4_ (LFP) cathodes paired with SD-based anodes. The LFP cathode mass loading was approximately 4.0 mg cm^−2^. The electrolyte consisted of 1 M LiPF_6_ in EC/DEC/EMC (1 : 1 : 1 by volume). The cells were assembled with an N/P ratio of approximately 1.2. No pre-lithiation process was applied to the full cells. The voltage window for full-cell operation was 2.5–4.2 V. Prior to electrochemical evaluation, the assembled full cells underwent two initial activation cycles at 0.1C for electrode/electrolyte conditioning. These early conditioning cycles were not included in the electrochemical analysis due to transient interfacial instability during initial cycling. Subsequently, two additional cycles at 0.1C were performed to establish a stabilized electrochemical baseline. Long-term cycling performance was then evaluated at 0.5C (1C = 170 mA g^−1^), and the capacity retention was calculated starting from this stabilized state.

### 2.5. Theoretical simulations

All density functional theory (DFT) calculations were performed using the DMol3 module in Materials Studio 2020. The exchange–correlation functional was described using the Generalized Gradient Approximation (GGA) with the BLYP functional. A double numerical plus polarization (DNP) basis set was employed. The convergence criteria for energy, maximum force, and maximum displacement were set to 1.0 × 10^−5^ Ha, 0.002 Ha Å^−1^, and 0.005 Å, respectively. To simulate the interfacial reactions, an amorphous Li_3_AlF_6_ surface model was constructed to represent the experimental group, while a crystalline Si(111) surface with an amorphous SiO_*x*_ overlayer served as the control group. The adsorption energies (*E*_ads_) were calculated as follows:

1 *E*_ads_ = *E*_total_ − (*E*_surface_ + *E*_molecular_)

## 3. Results and discussion

### 3.1. Structure and morphology characterization

As schematically illustrated in Fig. 1a, the defect-to-design strategy initiates with the selective chemical dealloying of spherical Si_10_Al_90_ alloy particles (∼5 µm, Fig. 1b). In this stage, HCl treatment removes the majority of the Al matrix to create a hierarchical SD framework. The SEM image of SD (Fig. 1b) confirms the formation of a well-developed porous structure, while the corresponding elemental mapping in Fig. S1 shows the uniform bulk distribution of residual Al within the SD framework. These metallic “defects” were subsequently repurposed into functional interfacial sites through kinetically controlled HF treatment. The SEM image of SD@AlF_3_ (Fig. 1b) reveals that the overall micro-scale morphology and particle size remain intact after etching with 5 wt% HF, ensuring the preservation of particle-scale integrity at the electrode level. However, comprehensive mapping and EDS statistics of the SD@AlF_3_-*x* series (Fig. S2 and S3) indicate a clear trend: as HF concentration increases from 2% to 8%, the F-content increases systematically while the O-content drops (Table S1). This trend reflects a progressive replacement of the native oxide by fluoride species. It is noted that the residual Al content is primarily determined by the precursor alloy composition and the HCl dealloying process, which leads to partial removal of the Al matrix while retaining a small amount of Al within the porous Si framework. Subsequent HF treatment mainly modulates the interfacial chemistry by thinning the native SiO_*x*_ layer and converting surface Al species into AlF_3_, without significantly altering the overall Al content (Table S1). Excessive HF treatment, however, may over-etch the Si framework and compromise structural stability. While XRD patterns of SD and SD@AlF_3_ (Fig. 1c) show only the characteristic reflections of cubic diamond Si (JCPDS #27-1402),^29^ the preservation of crystalline integrity across the SD@AlF_3_-*x* series (Fig. S4) underscores that the HF treatment is strategically confined to the surface rather than attacking the structural core.

The surface bonding evolution was meticulously tracked *via* Fourier-transform infrared (FTIR) spectroscopy. In Fig. 1d, the SD sample exhibits intense vibrations at ∼1080, 781, and 457 cm^−1^, corresponding to the Si–O–Si stretching and bending modes of a thick native SiO_*x*_ shell.^30^ Upon optimized HF treatment (SD@AlF_3_), these features are significantly attenuated, replaced by a prominent absorption band at ∼635 cm^−1^ assigned to Al–F stretching.^31^ The FTIR spectra of the entire series (Fig. S5) further demonstrate this transition, showing that the Al–F signal reaches a plateau at 5–8 wt% HF. This interfacial chemical remodeling arises from following competing HF-driven reactions (eqn (2)–(4)).^22,32^ While reactions (2)–(4) are beneficial for thinning the oxide and generating the AlF_3_ catalyst, excessive HF concentrations (8 wt%) trigger a parasitic reaction with the crystalline Si framework (eqn (5)).^22^ This over-etching leads to ligament thinning and local structural fragility observed in SD@AlF_3_-8 (Fig. S2).

2 SiO_2_(s) + 6HF(aq) → H_2_SiF_6_(aq) + 2H_2_O(l)

3 Al_2_O_3_(s) + 6HF(aq) → 2AlF_3_(s) + 3H_2_O(l)

4 2Al(s, in Al–Si alloy) + 6HF(aq) → 2AlF_3_(s) + 3H_2_(g)

5 Si(s) + 6HF(aq) → H_2_SiF_6_(aq) + 2H_2_(g)

To gain deeper insights into the surface electronic states, XPS was conducted. The survey spectra (Fig. 1f and S6) reveal a significant increase in F 1s and Si 2p intensities at the expense of O 1s, confirming the systematic thinning of the SiO_*x*_ shell. High-resolution Si 2p spectra (Fig. 1g and S7) further resolve this by showing a transition from Si–O (∼102.7 eV) to Si–Si dominance (∼98.6 eV). The calculated area ratio *S*_Si–O_/*S*_Si–Si_ (Fig. S8) reveals that the SiO_*x*_ thickness is precisely tailored by the HF kinetics. The Al 2p spectra further demonstrate the conversion of residual Al (Al–Si alloy/oxide) into a dominant Al–F coordination (∼76.0 eV), corroborated by the F 1s peak at ∼686.0 eV.^32^

The structural consequence of this surface engineering is a profound change in textural and interfacial properties. N_2_ adsorption–desorption isotherms (Fig. 1e and S9) indicate a progressive increase in BET specific surface area from 70.1 m^2^ g^−1^ (SD) to 143.7 m^2^ g^−1^ (SD@AlF_3_-8). However, pore size distribution analysis (Fig. S10 and Table S2) shows that SD@AlF_3_ achieves the optimal balance with the highest mesopore fraction (58.0%), whereas SD@AlF_3_-8 suffers from pore coalescence and macropore formation. As evidenced by contact angle measurements using carbonate-based electrolyte (1 M LiPF_6_ in EC/DEC (1 : 1 by volume) with 10 wt% FEC additive) (Fig. S11), the SD@AlF_3_ surface exhibits the lowest angle (7.6°) compared to SD (17.7°), SD@AlF_3_-2 (13.6°) and SD@AlF_3_-8 (10.6°), indicating a superior electrolyte-philic interface that facilitates homogeneous ion transport.

**Fig. 2:**
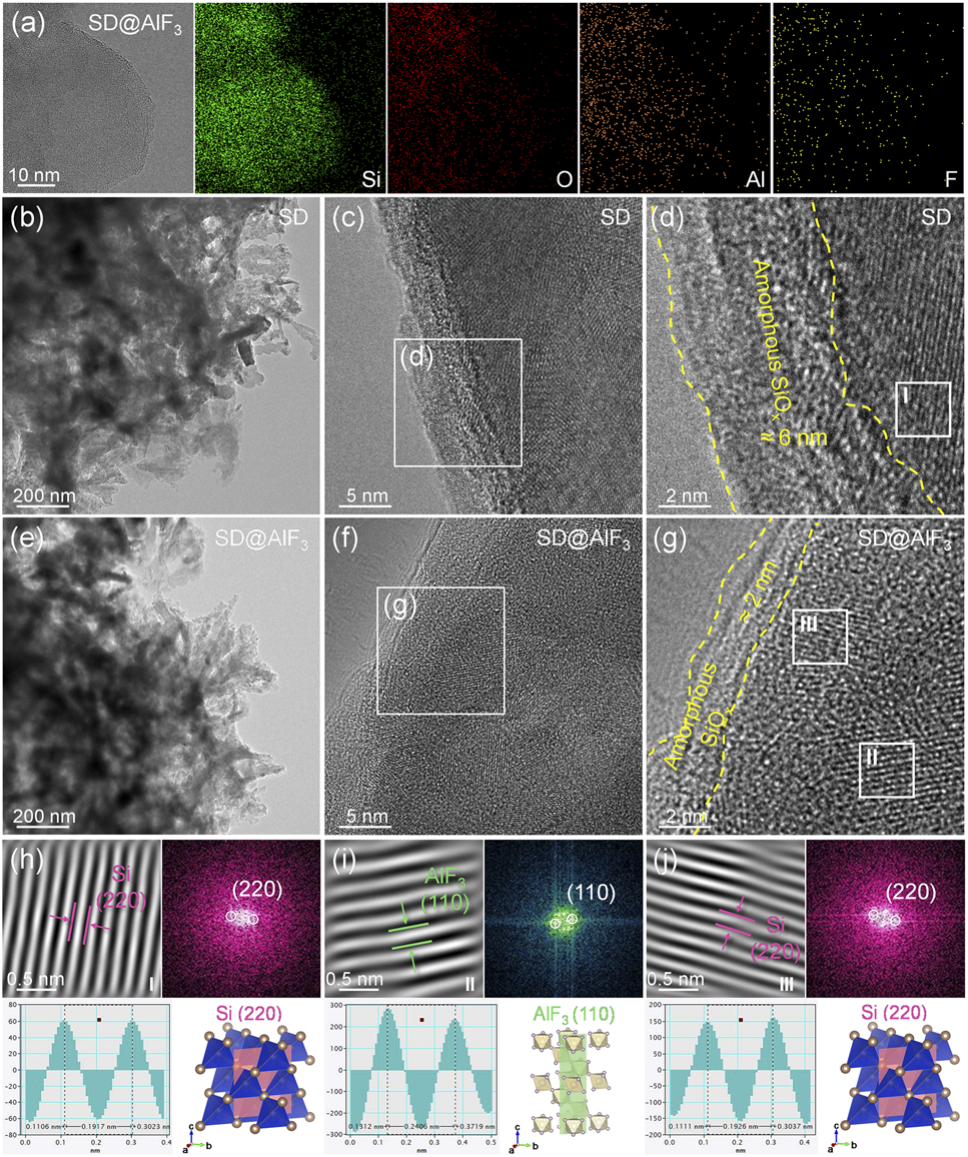
Microstructure and interfacial crystallography of SD and SD@AlF_3_. (a) EDS elemental mapping of SD@AlF_3_. (b–d) TEM/HRTEM images of SD. (e–g) TEM/HRTEM images of SD@AlF_3_. (h–j) FFT patterns and corresponding IFFT images from the regions marked in (d) and (g).

The nanoscale interfacial architecture was systematically investigated to clarify how HF treatment regulates the thickness and composition of the amorphous surface layer. EDS elemental mapping (Fig. S12a) first reveals the interfacial enrichment of residual Al in SD, accompanied by a pronounced O signal outlining the particle surfaces, indicating the presence of a relatively thick amorphous SiO_*x*_ layer. This compositional distribution suggests that a fraction of residual Al migrates toward the interface during the dealloying and subsequent aqueous washing processes, resulting in an Al-modified surface oxide. Such intrinsic enrichment provides a mechanistic basis for the subsequent *in situ* fluorination, as these pre-positioned Al “defects” can serve as reactive sites. Consistent with this observation, TEM images of SD (Fig. 2b) confirm the formation of interconnected dendritic ligaments. HRTEM analysis of representative ligament surfaces (Fig. 2c, d and h) further identifies a crystalline Si core encapsulated by a dense amorphous SiO_*x*_ shell with a thickness of ∼6 nm, in agreement with the compositional mapping as well as the FTIR and XPS results discussed above.

For SD@AlF_3_, EDS mapping (Fig. 2a) demonstrates a homogeneous distribution of Si, O, Al, and F throughout the interfacial region, confirming the successful incorporation of fluorine species and the uniform dispersion of Al-containing components. This uniform elemental distribution indicates that the fluorination process occurs *in situ* at the interface rather than forming isolated precipitates. The dendritic architecture is well preserved after HF treatment, as evidenced by TEM imaging (Fig. 2e). However, the interfacial structure undergoes a substantial transformation. HRTEM images (Fig. 2f, g and j) reveal that the amorphous shell is uniformly thinned to an approximate thickness of ∼2 nm, corresponding to the best-performing interfacial conditions within the investigated HF-treatment window. The magnified region III in Fig. 2g (Fig. 2j) displays clear lattice fringes corresponding to crystalline Si, consistent with the core structure observed in pristine SD (Fig. 2h). Further structural analysis *via* FFT of region II in Fig. 2g, together with the corresponding IFFT image, can be indexed to the AlF_3_(110) planes with a *d*-spacing of 0.24 nm, unambiguously confirming the *in situ* formation of AlF_3_ nanocatalysts (Fig. 2i). These results collectively demonstrate the construction of an ultrathin SiO_*x*_ interfacial layer decorated with homogeneously embedded AlF_3_ nanocrystallites, forming an integrated AlF_3_-modified SiO_*x*_ bilayer architecture. This integrated interfacial structure is designed to balance three competing requirements: (i) providing sufficient mechanical stiffness to restrain Si expansion, (ii) maintaining an ultrathin profile to minimize Li^+^ diffusion resistance, and (iii) offering catalytic sites to regulate SEI formation.

The importance of achieving a balanced interfacial thickness is further illustrated by comparative HRTEM analysis of the SD@AlF_3_-*x* series (Fig. S12b and c). At a low HF concentration (SD@AlF_3_-2), the remaining amorphous layer (∼4 nm) is relatively thick, which is expected to hinder interfacial ion transport. Conversely, at high HF concentrations (SD@AlF_3_-8), the surface layer is reduced to ∼1 nm, rendering the Si ligaments more susceptible to mechanical instability and surface cracking under repeated volume fluctuations.^20,33–36^ These observations indicate that an appropriate interfacial thickness is required to balance interfacial accessibility and structural robustness.

### 3.2. Electrochemical performance

**Fig. 3:**
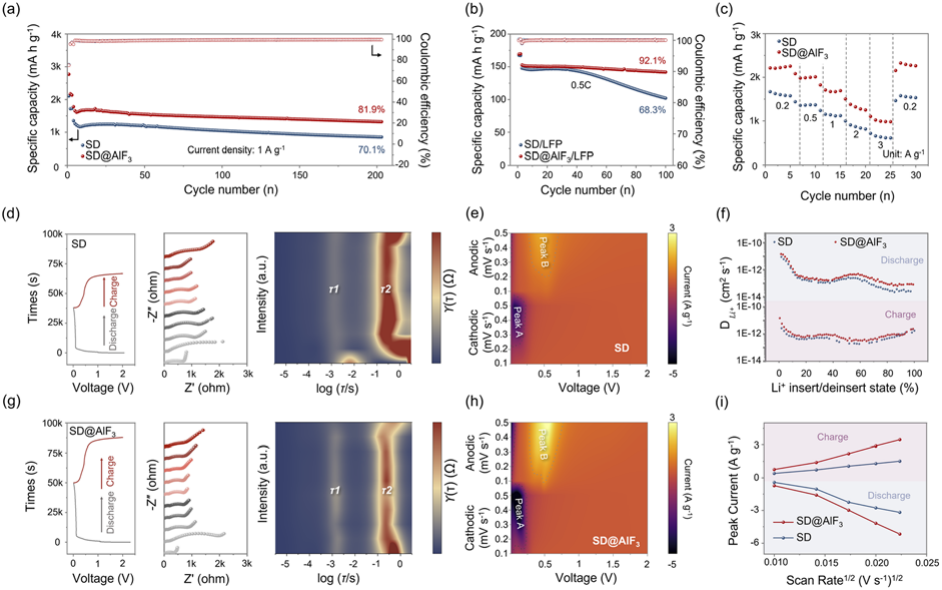
Electrochemical performance and interfacial kinetics of SD and SD@AlF_3_ electrodes. (a) Cycling stability and coulombic efficiency at 1 A g^−1^. (b) Cycling performance of SD and SD@AlF_3_ in full-cell configuration using a commercial LFP cathode. (c) Rate capability at current densities from 0.2 to 3 A g^−1^. (d and g) Potential-resolved EIS Nyquist plots and corresponding DRT analysis. (e and h) Scan-rate-dependent CV contour maps across varied scan rates. (f) Comparison of Li^+^ diffusion coefficients (*D*_Li^+^_) derived from GITT. (i) Linear fitting of peak current (*i*_p_) *versus* the square root of scan rate (*ν*^1/2^) for cathodic and anodic processes.

The electrochemical storage performance validates the defect-to-design strategy, illustrating how the precisely tailored surface chemistry translates into superior lithium storage. As shown in Fig. 3a, the long-term cycling stability at 1 A g^−1^ follows a distinct volcano trend relative to HF concentration. The SD@AlF_3_ electrode delivers the most robust performance, retaining 81.9% of its capacity after 200 cycles, significantly outperforming SD (70.1%) and the over-etched SD@AlF_3_-8 (68.6%) (Fig. S13 and S14). Analysis of the initial GCD profiles (Fig. S15) and the initial coulombic efficiency (ICE) histograms (Fig. S14) reveals a nuanced correlation between the surface area and interfacial chemistry. Notably, SD@AlF_3_-2 exhibits the highest ICE of 79.7%, which arises from a favorable balance between interfacial chemistry and surface area: the partial removal of the SiO_*x*_ shell suppresses the irreversible lithium consumption associated with SiO_*x*_ reduction, while the increase in the surface area remains sufficiently moderate to limit parasitic electrolyte decomposition.^20^ For the optimized SD@AlF_3_, the further thinning of the SiO_*x*_ shell minimizes interfacial resistance, though the concurrently increased surface area introduces a slight trade-off in ICE. The ICE is governed by multiple coupled factors, including irreversible SiO_*x*_ reduction, electrolyte consumption related to surface area, and initial SEI formation processes.

To further evaluate the practical applicability of the SD@AlF_3_ electrode, full-cell configurations were assembled using commercial LiFePO_4_ (LFP) cathodes. Unlike half-cell systems that employ excess lithium reservoirs, full cells provide a more realistic assessment of electrode performance under limited lithium inventory conditions. As shown in Fig. 3b, the SD@AlF_3_‖LFP full cell delivers significantly enhanced cycling stability compared to the SD-based counterpart. Importantly, the SD@AlF_3_ full cell retains 92.1% of its capacity after 100 cycles at 0.5C, whereas the SD-based cell exhibits rapid capacity decay (68.3% retention under identical conditions). This pronounced difference confirms that the optimized interfacial structure effectively translates into improved electrochemical performance at the device level, highlighting its practical relevance for high-energy-density lithium-ion batteries.

CV was performed to verify whether the surface modification alters the fundamental lithiation mechanism (Fig. S16). Characteristic redox peaks corresponding to the stepwise (de)alloying of crystalline silicon are consistently observed across all samples, confirming that the AlF_3_-modified interface regulates ion flux without perturbing the intrinsic redox chemistry.^37,38^ This is further supported by the high reversibility shown in subsequent GCD curves (Fig. S17). Notably, the superior electrochemical performance of SD@AlF_3_ is benchmarked against recently reported HF-etched silicon anodes in a radar plot (Fig. S20 and Table S3), highlighting its overall competitiveness.^22,33,38–42^

The kinetic advantages of the engineered interface are particularly pronounced in rate capability tests (Fig. 3c). SD@AlF_3_ consistently delivers higher reversible capacities across all current densities (0.2–3 A g^−1^), retaining ∼1015 mAh g^−1^ at the maximum rate, whereas SD and SD@AlF_3_-8 show significantly lower high-rate capacity (Fig. S18). These enhanced kinetics are corroborated by the apparent Li^+^ diffusion coefficient (*D*_Li^+^_) derived from GITT measurements (Fig. 3f and S19). SD@AlF_3_ exhibits the highest *D*_Li^+^_ throughout the (de)lithiation process, which is attributed to the synergistic combination of an ultrathin surface layer and pre-positioned catalyst sites that facilitate rapid interfacial ion transport.

To further decouple the interfacial resistance, potential-resolved EIS and the corresponding distribution of relaxation times (DRT) analysis were performed (Fig. 3d and g). In the DRT plots, two characteristic peaks are resolved: *τ*_1_ (10^−2.5^ s) and *τ*_2_ (10^−0.5^ s), assigned to Li^+^ migration through the SEI and the charge-transfer process, respectively. Notably, the SD@AlF_3_ electrode retains significantly lower resistance across all potential stages compared to SD. This suggests that the optimized interface facilitates a more efficient ion-conduction pathway (later identified as a Li–Al–F network in Fig. 6), effectively lowering the kinetic barriers for lithium storage. This superiority is further substantiated by variable-scan-rate CV contour plots (Fig. 3e and h). Quantitative analysis of the peak current (*i*_p_) *versus* the square root of scan rate (*ν*^1/2^) (Fig. 3i) reveals a substantially higher slope for SD@AlF_3_ compared to SD, confirming accelerated charge-transfer and diffusion kinetics enabled by the catalyst-modified interface, even under high current loads.

### 3.3. Electrochemical mechanism

**Fig. 4:**
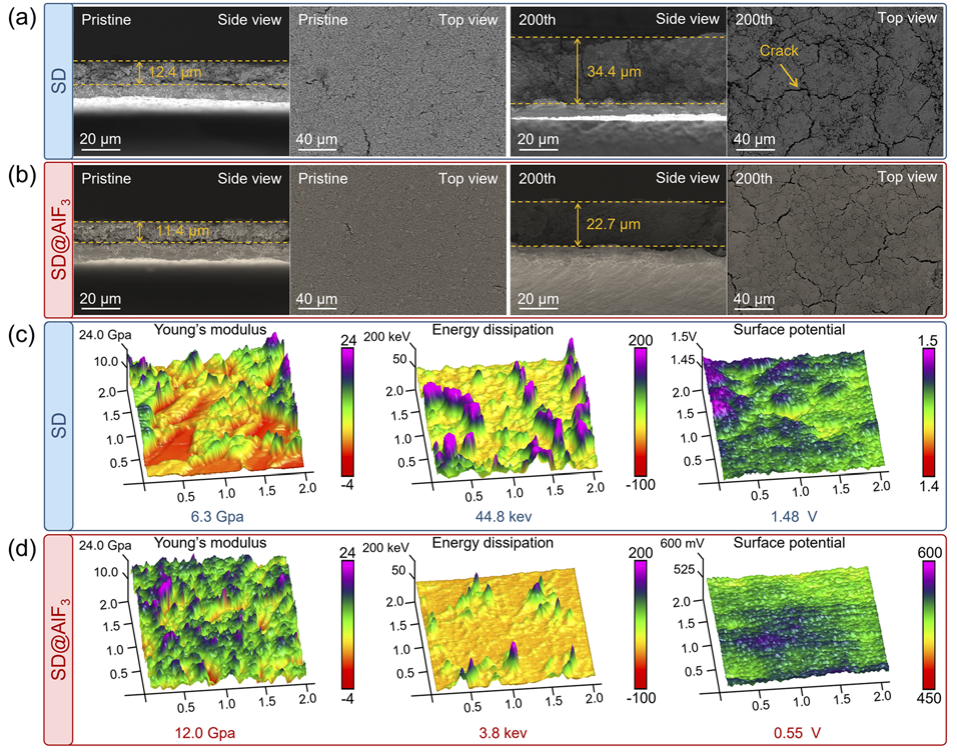
Post-cycling mechanical analysis. (a and b) SEM cross-sectional and top-view images of cycled SD (a) and SD@AlF_3_ (b) electrodes. (c and d) AFM nanomechanical mapping of cycled SD (c) and SD@AlF_3_ (d), including Young's modulus, energy dissipation, and surface potential.

Interfacial stabilization confers exceptional resistance against electrode-level expansion. Cross-sectional SEM after 200 cycles (Fig. 4a) reveals that the SD electrode suffers from catastrophic swelling of ∼177.4% (from 12.4 to 34.4 µm). In stark contrast, SD@AlF_3_ expands only 99.1% (from 11.4 to 22.7 µm), representing an approximately 80% suppression compared to that of SD. Comparative analysis (Fig. S21) confirms that, while SD@AlF_3_-2 and SD@AlF_3_-8 undergo severe degradation, the optimized SD@AlF_3_ maintains superior structural integrity. This macroscopic robustness is corroborated by post-cycling EIS (Fig. S22 and Table S4), where SD@AlF_3_ exhibits the lowest *R*_ct_ (617.4 Ω) and *R*_SEI_ (114.1 Ω) among all samples after 200 cycles. In contrast, SD shows a dramatic increase in both *R*_ct_ (1615.6 Ω) and *R*_SEI_ (124.5 Ω), indicating severe interfacial degradation. Notably, SD@AlF_3_-8 also suffers from significant impedance growth, consistent with its over-etched structure. These quantitative results confirm that the optimized SD@AlF_3_ electrode exhibits the most stable interfacial kinetics during prolonged cycling.

Nanoscale stability was further probed *via* AFM. SD@AlF_3_ displays a lower surface roughness (*R*_a_ = 51.8 nm) and a more homogeneous topography than SD (*R*_a_ = 86.8 nm, Fig. S23), indicating the formation of a denser and more uniform SEI.^43,44^ PeakForce quantitative nanomechanical mapping (QNM, Fig. 4c and d) reveals that the SEI on SD@AlF_3_ possesses an exceptionally high Young's modulus of 12.0 GPa, nearly double that of the SEI on SD (6.3 GPa). Such stiffness is critical for resisting the lithiation-induced hoop stress and preventing interfacial delamination. Additionally, the significantly lower energy dissipation of SD@AlF_3_ (3.8 keV *vs.* 44.8 keV for SD) underscores the formation of a highly elastic and compact SEI framework.^44,45^

Kelvin probe force microscopy (KPFM) was utilized to evaluate electrical passivation (Fig. 4c and d). Unlike the erratic, high surface potential (contact potential difference, CPD) of cycled SD (averaging 1.48 V), SD@AlF_3_ displays a remarkably low and uniform CPD of 0.55 V. This signifies enhanced electronic passivation that limits electron tunneling from the electrode and suppresses persistent electrolyte reduction. Combined with the lower surface adhesion force (64 nN *vs.* 202 nN for SD, Fig. S24), these results collectively demonstrate that the catalyst-modified surface orchestrates the evolution of an ideal SEI, which is mechanically rigid, electronically insulating, and structurally robust.^46^

**Fig. 5:**
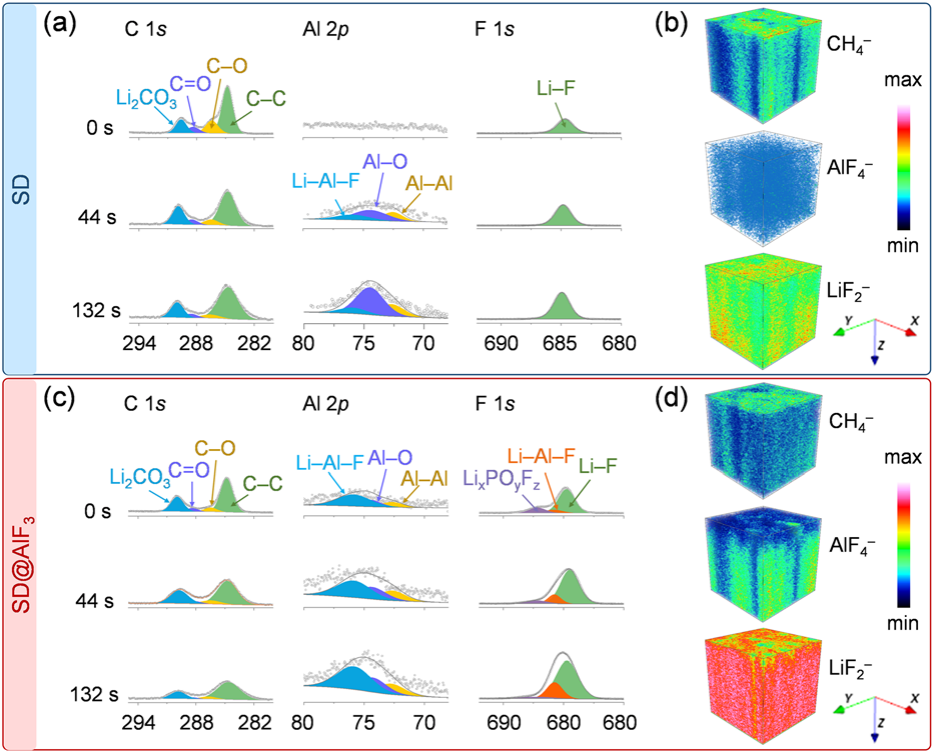
Interfacial mechanism. (a and c) Depth-profiled XPS spectra of cycled SD (a) and SD@AlF_3_ (c) electrodes. (b and d) 3D TOF-SIMS ion maps of cycled SD (b) and SD@AlF_3_ (d), showing the distribution of CH_4_^−^, AlF_4_^−^, and LiF_2_^−^ fragments.

To unveil the mechanistic origin of the superior electrochemical stability, depth-profiled XPS and 3D TOF-SIMS were employed to interrogate the chemical architecture of the cycled SEI (Fig. 5). For the SD electrode, XPS spectra reveal a thick outer layer of organic decomposition products that persist throughout the sputtering process (Fig. 5a). Notably, trace Li–Al–F species (∼75.7 eV in Al 2p) are also detected in SD; however, their presence originates from the uncontrolled spontaneous reaction between residual metallic Al and HF generated by LiPF_6_ decomposition in the electrolyte, yielding a spatially heterogeneous and fragmented interphase.^47^ In stark contrast, SD@AlF_3_ exhibits a significantly thinner organic-rich layer and a uniform intense enrichment of Li–Al–F species across all depths (Fig. 5c). This structured interphase is derived from the *in situ* electrochemical transformation of the precisely engineered AlF_3_ precursor during initial cycling (eqn (6)).^32,48,49^

6 2AlF_3_(s) + 3Li^+^ + 3e^−^ → Li_3_AlF_6_(s) + Al(s)

Compared to traditional external coating techniques, such as magnetron sputtering or atomic layer deposition,^32^ this *in situ* chemical transformation strategy ensures that the AlF_3_ precursor is chemically anchored to the Si ligaments, enabling the formation of a more resilient and integrated Li–Al–F network during repeated (de)lithiation. This structured interphase is vividly visualized by 3D TOF-SIMS imaging (Fig. 5b and d), where inorganic framework ions (AlF_4_^−^ and LiF_2_^−^) are uniformly distributed throughout the SEI volume, while organic fragments (CH_4_^−^) are largely confined to the surface. Crucially, the evolved Li–Al–F phase is reported to possess high ionic conductivity (∼10^−6^ to 10^−4^ S cm^−1^), which effectively lowers the interfacial charge-transfer barrier, while its intrinsic low electronic conductivity ensures essential passivation against parasitic electrolyte reduction.^32,48,49^

**Fig. 6:**
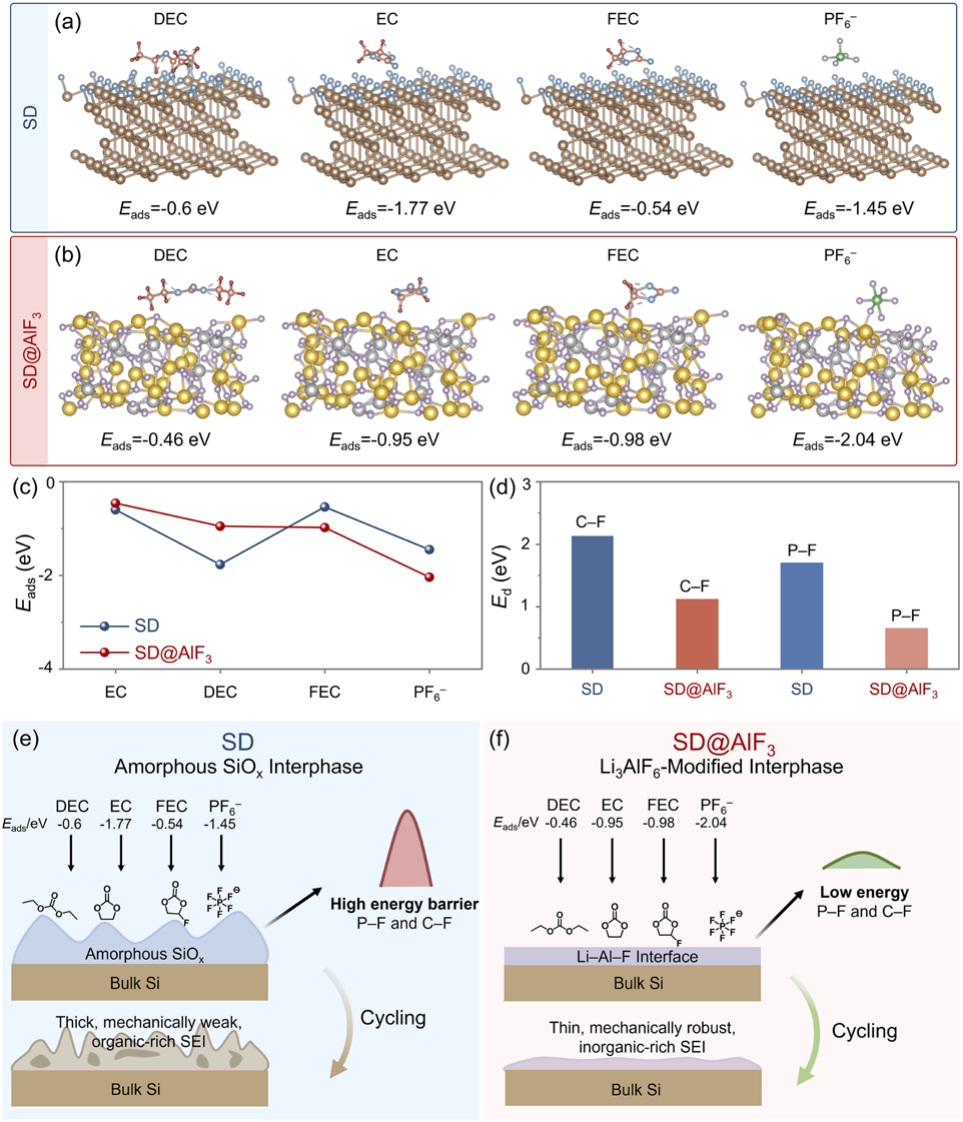
DFT simulations. (a and b) DFT-optimized adsorption configurations of EC, DEC, FEC, and PF_6_^−^ on the Si@SiO_*x*_ and Li_3_AlF_6_ interface models. (c and d) Calculated adsorption energies of the four electrolyte species and the dissociation energy barriers for the C–F (in FEC) and P–F (in PF_6_^−^) bonds on the two interfaces. (e and f) Schematic illustrating the working mechanisms of the amorphous SiO_*x*_ interphase and the Li_3_AlF_6_-modified interphase.

The catalytic function of this Li–Al–F network was quantified *via* DFT simulations (Fig. 6a–d). The modeled Li_3_AlF_6_ interface (representing the evolved interphase) exhibits a significantly stronger adsorption energy (*E*_ads_) for PF_6_^−^ and FEC compared to the native Si@SiO_*x*_ surface. Most importantly, it catalytically lowers the dissociation energy (*E*_d_) barriers for both the P–F bond in PF_6_^−^ and the C–F bond in FEC, facilitating the preferential formation of an inorganic-rich SEI through the following pathways (eqn (7) and (8)).^50^ For clarity, the terms “stronger” and “weaker” interactions refer to relative adsorption strengths of the same molecule across different interfaces.

7 PF_6_^−^ + 3Li^+^ + 2e^−^ → 3LiF + PF_3_

8
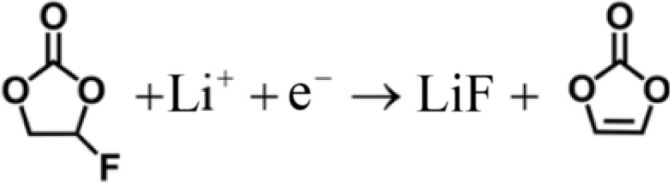


Complementary analysis of the Si 2p XPS spectra and Si^−^ TOF-SIMS ion maps (Fig. S25) confirms that this robust inorganic shield provides superior electronic passivation of the silicon skeleton. As schematically illustrated in Fig. 6e and f, the defect-to-design strategy fundamentally reconfigures the interfacial evolution pathway. While the native SD surface, with high dissociation barriers, leads to the chaotic growth of a thick, mechanically weak, and organic-dominated SEI, the Li–Al–F network derived from repurposed residual Al provides a functional template to orchestrate an ideal SEI that is thin, mechanically rigid, ionically transparent, and electronically insulating. This paradigm effectively tames the interfacial instability of SD, ensuring sustainable and high-capacity lithium storage. To avoid overinterpretation, the calculated dissociation barriers are not intended to provide a direct quantitative correlation with macroscopic electrochemical metrics. Instead, they serve as a descriptor for the catalytic tendency toward inorganic SEI formation on the Li–Al–F interface, which operates in synergy with the structural buffering effect of the SiO_*x*_ layer. The experimentally observed enrichment of LiF/Li–Al–F species (XPS and TOF-SIMS) supports this interpretation. It should be emphasized that the remarkable performance enhancement of the SD@AlF_3_ electrode in this study arises from the synergistic combination of interfacial structural optimization and catalytic effects induced by HF etching. On one hand, the controlled thinning of the SiO_*x*_ shell shortens the ion transport pathways and physically mitigates the volume expansion stress; on the other hand, the *in situ* formed Li–Al–F network facilitates the adsorption and catalytic decomposition of FEC and PF_6_^−^, guiding the formation of a highly rigid inorganic-rich SEI. Although the gradient experiments of the SD@AlF_3_-*x* series and the DRT analyses have qualitatively confirmed the significant contributions from both mechanisms, precise numerical quantification of their relative contributions remains challenging because these two interfacial variables cannot be independently decoupled *via* the current synthesis strategy.

## 4. Conclusions

In conclusion, we have demonstrated a defect-to-design interfacial engineering strategy that successfully repurposes intrinsic residual Al in dealloyed porous silicon into an active regulator of SEI formation. By controlling HF etching kinetics, the native SiO_*x*_ layer is tailored to a favorable thickness of ∼2 nm within the investigated SD@AlF_3_-*x* series, while residual Al is transformed *in situ* into nanometric AlF_3_ catalyst precursors. This engineered interface dynamically evolves into a conductive Li–Al–F network during cycling, which preferentially anchors electrolyte components and catalytically lowers the dissociation energy barriers of PF_6_^−^ and FEC. This orchestrated catalytic process guides the formation of an ideal SEI that is thin, inorganic-rich, and mechanically robust, with a high Young's modulus of 12.0 GPa. Consequently, SD@AlF_3_ retains 81.9% of its capacity after 200 cycles (half-cell) and shows a decrease in electrode swelling from 177.4% to 99.1%. The SD@AlF_3_‖LFP full cell further retains 92.1% of its capacity after 100 cycles at 0.5C, demonstrating the practical relevance of the strategy. This work not only uncovers the previously neglected chemical reactivity of intrinsic defects in Al–Si dealloying systems but also establishes a scalable and effective paradigm for leveraging inherent material features to engineer multifunctional interphases for high-energy-density batteries. Conceptually, this residual-Al-mediated defect-to-design strategy may be extended to other Al-containing alloy anodes, such as Sn–Al and Ge–Al systems, although the alloy composition, dealloying kinetics, and fluorination window should be re-optimized for each material system.

## Author contributions

Xiang Wang: conceptualization, investigation, methodology, writing – original draft. Xiaofan Liu: investigation, data curation. Yinjiang Du: investigation, formal analysis. Yue Lu: data curation, visualization. Xinyue Dong: methodology, validation. Wenqing Ma: resources, supervision, writing – review and editing. Xiangping Chen: resources, methodology. Jiang Yin: conceptualization, supervision, writing – review and editing. Yahui Yang: formal analysis, validation. Yanqing Lai: investigation, resources. Xiongwei Wu: methodology, supervision. Lishan Yang: conceptualization, supervision, writing – review and editing, funding acquisition.

## Conflicts of interest

There are no conflicts to declare.

## Supplementary Material

SC-OLF-D6SC04327E-s001

## Data Availability

The data supporting this article are available in the main text and the supplementary information (SI). Supplementary information: detailed morphological, structural, and spectroscopic characterization (SEM, TEM, XRD, XPS, FTIR, and AFM) and electrochemical performance data (cycling stability, rate capability, CV, GITT, and EIS) for all electrodes. See DOI: https://doi.org/10.1039/d6sc04327e.
