# Supplementary material for: Catalytic interfacial construction of a Li–Al–F-rich SEI for robust silicon dendrite anodes
Source: Chem Sci. 2026 Jul 20;17(36):17339–47. doi: 10.1039/d6sc04327e (PMC13417788; doi:10.1039/d6sc04327e)
Supplement: Supplementary file 1 [file SC-017-D6SC04327E-s001.pdf]

Supplementary Information (SI) for Chemical Science.
This journal is © The Royal Society of Chemistry 2026

Supporting Information

Catalytic interfacial construction of Li-Al-F-rich SEI for robust silicon dendrite anodes

Xiang Wang[a], Xiaofan Liu[a], Yinjiang Du[a], Yue Lu[b], Xinyue Dong[b], Wenqing Ma[b,*], Xiangping Chen[a], Jiang Yin[a,*], Yahui Yang[a], Yanqing Lai[c], Xiongwei Wu[a], Lishan Yang[a,*]

[a] *College of Chemistry and Chemical Engineering, Hunan Normal University, Changsha 410081, China*
[b] *Institute for Advanced Interdisciplinary Research (iAIR), Collaborative Innovation Center of Technology and Equipment for Biological Diagnosis and Therapy in Universities of Shandong, Shandong Key Laboratory of Functional Materials for Integrated Lithium Niobate Photonics, Core Research Facilities, School of Chemistry and Chemical Engineering, University of Jinan, Jinan 250022, China*
[c] *School of Metallurgy and Environment, Central South University, Changsha 410083, China*

*Corresponding author.
E-mail address: ifc_mawq@ujn.edu.cn (W. Ma), yinjiang@hunnu.edu.cn (J. Yin) and lsyang@hunnu.edu.cn (L. Yang).

The supporting information includes 30 pages, 25 figures, and 4 tables.

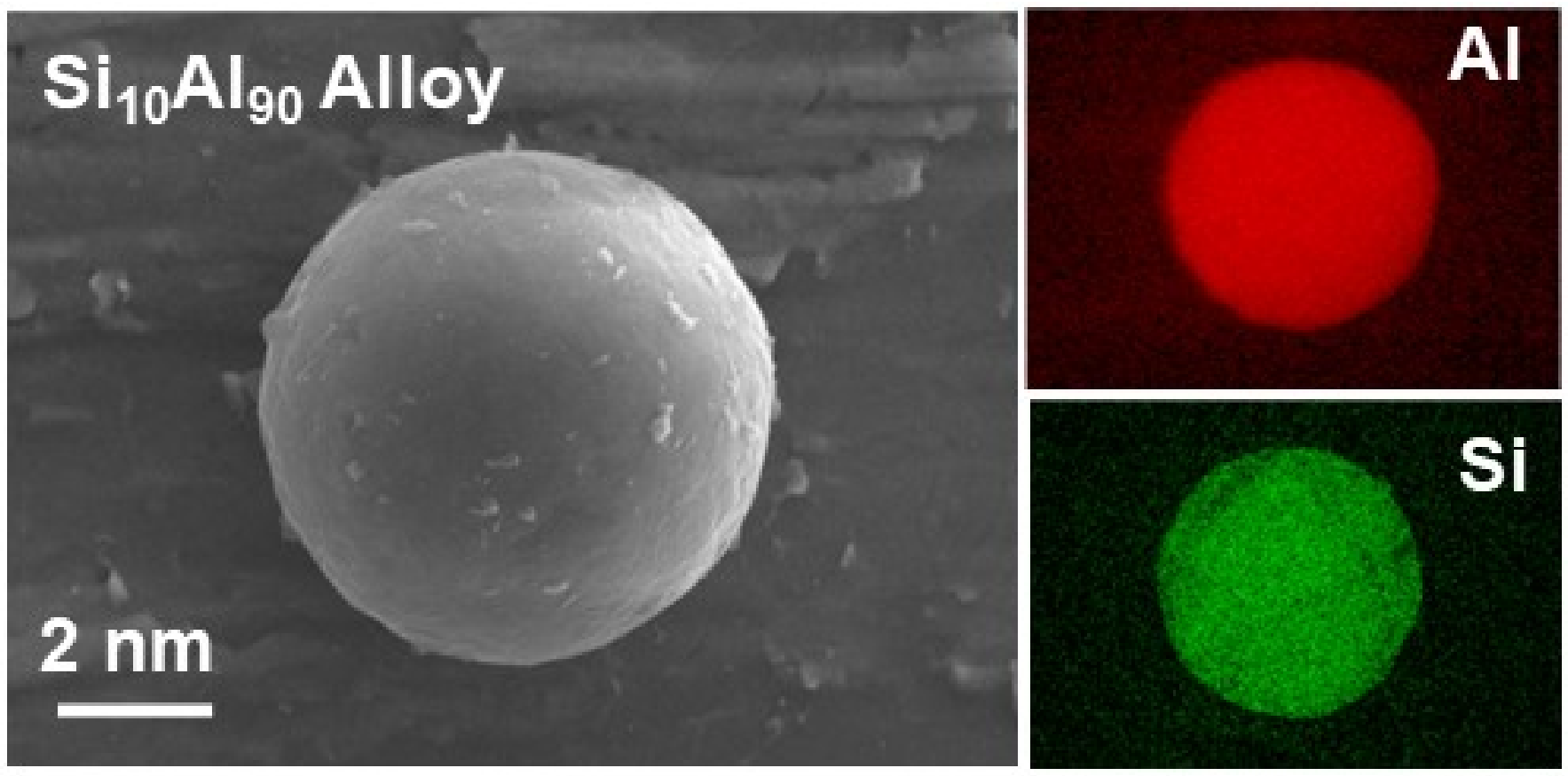

Fig. S1. SEM image and corresponding EDS elemental mapping (Si and Al) of the pristine $Si_{10}Al_{90}$ alloy.

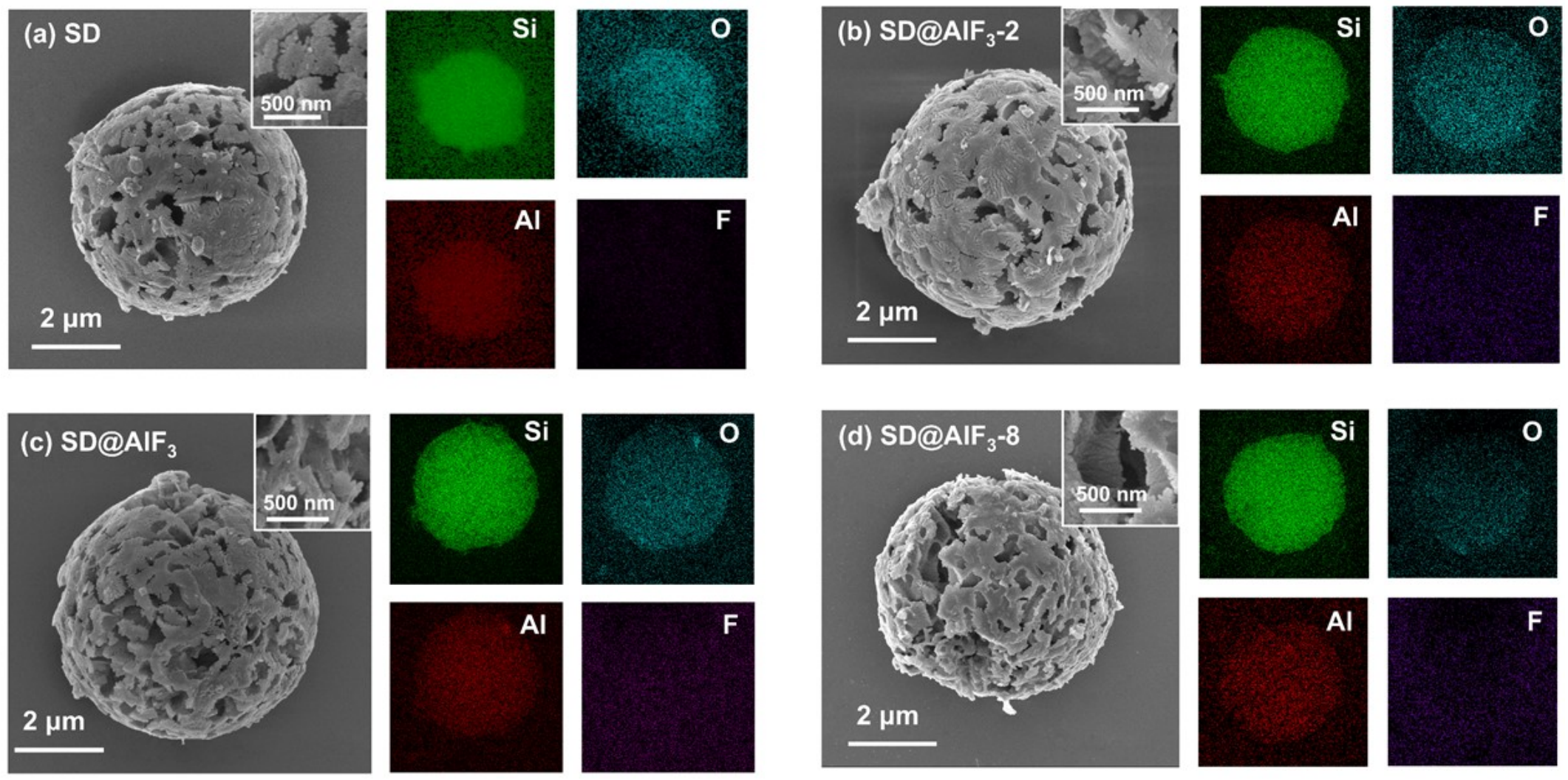

Fig. S2. SEM images and EDS elemental mapping (Si, O, Al, and F) of (a) SD, (b) SD@AlF_3-2, (c) SD@AlF_3, and (d) SD@AlF_3-8.

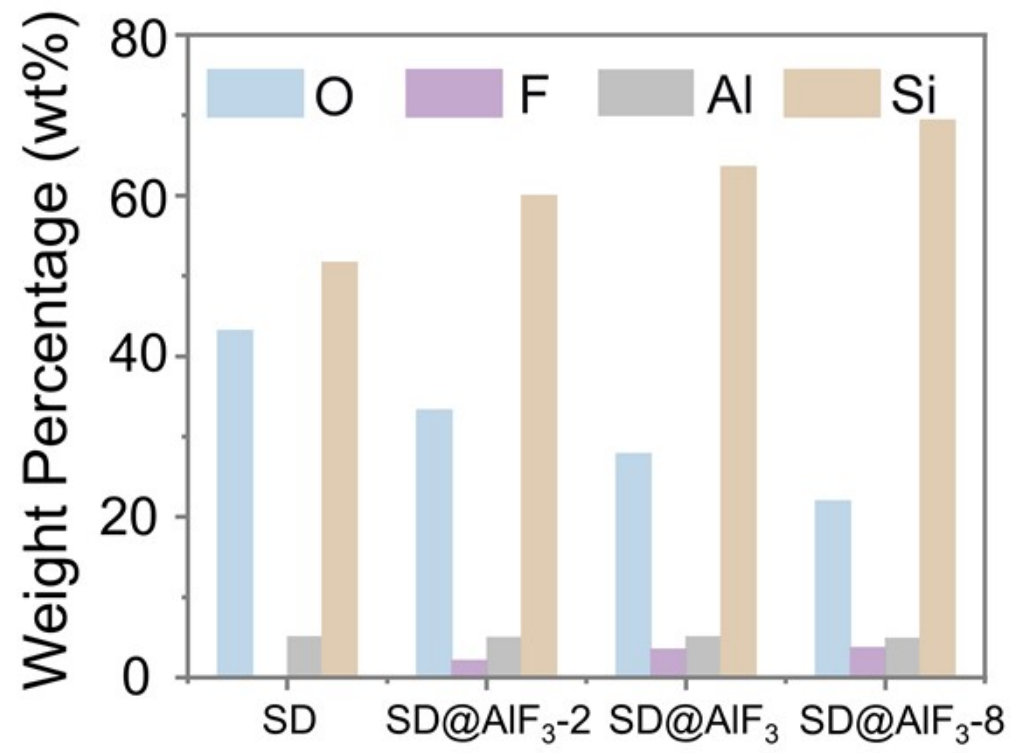

Fig. S3. Statistical histogram of elemental content (wt%) for SD, SD@AlF_3-2, SD@AlF_3, and SD@AlF_3-8 derived from EDS analysis.

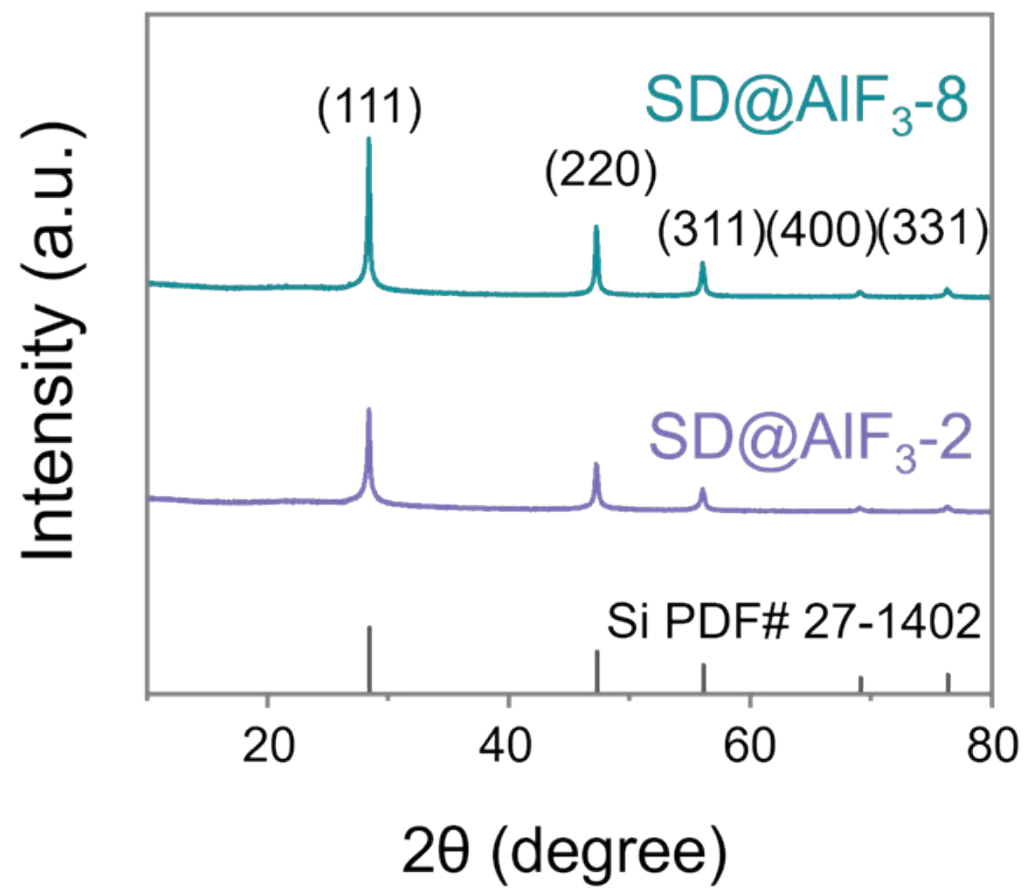

Fig. S4. XRD patterns of SD@AlF_3-2 and SD@AlF_3-8.

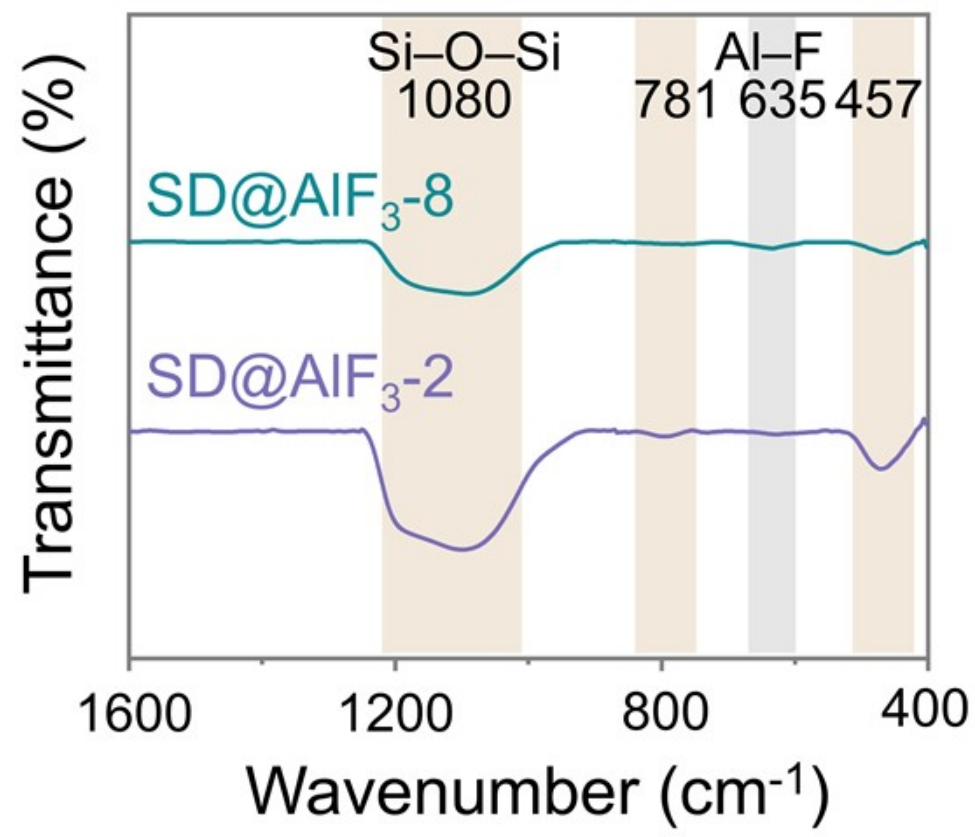

Fig. S5. FTIR spectra of SD@AlF_3-2 and SD@AlF_3-8.

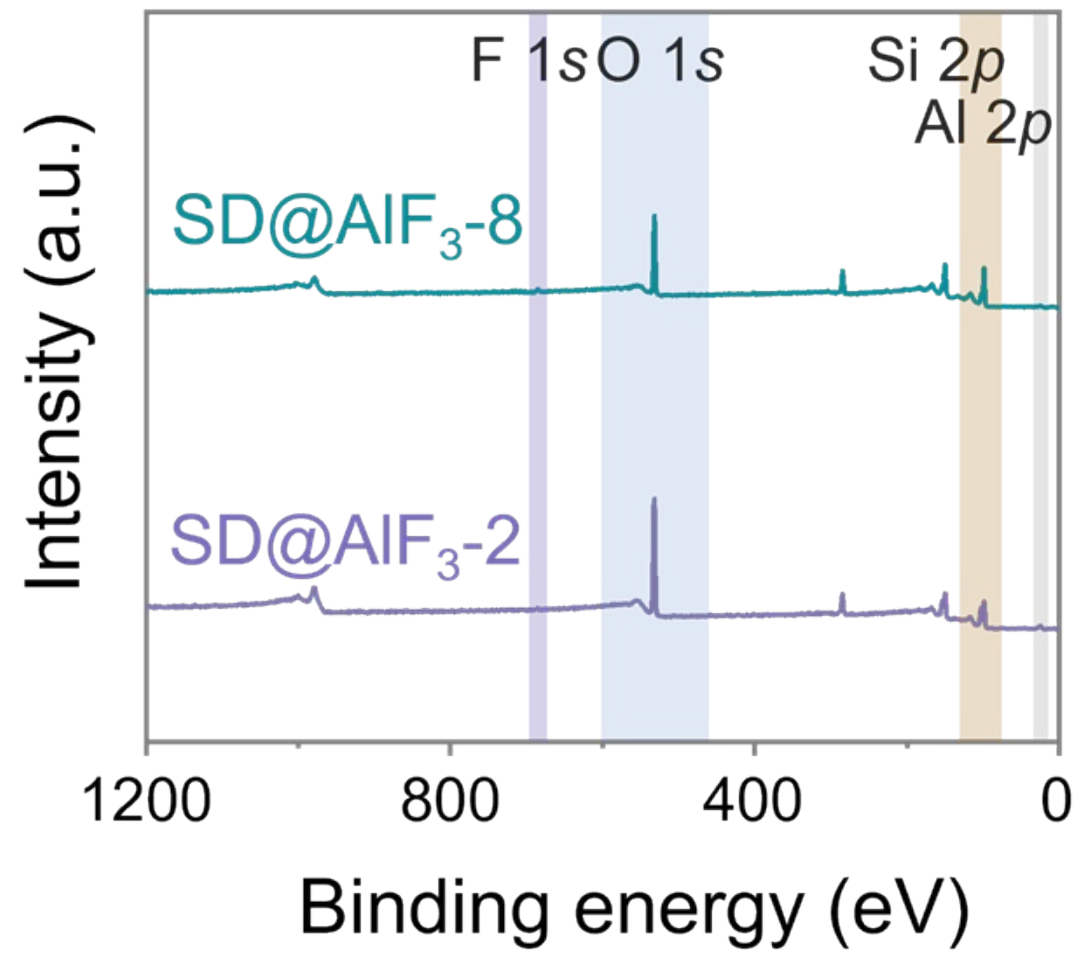

Fig. S6. XPS survey spectra of SD@AlF_3-2 and SD@AlF_3-8.

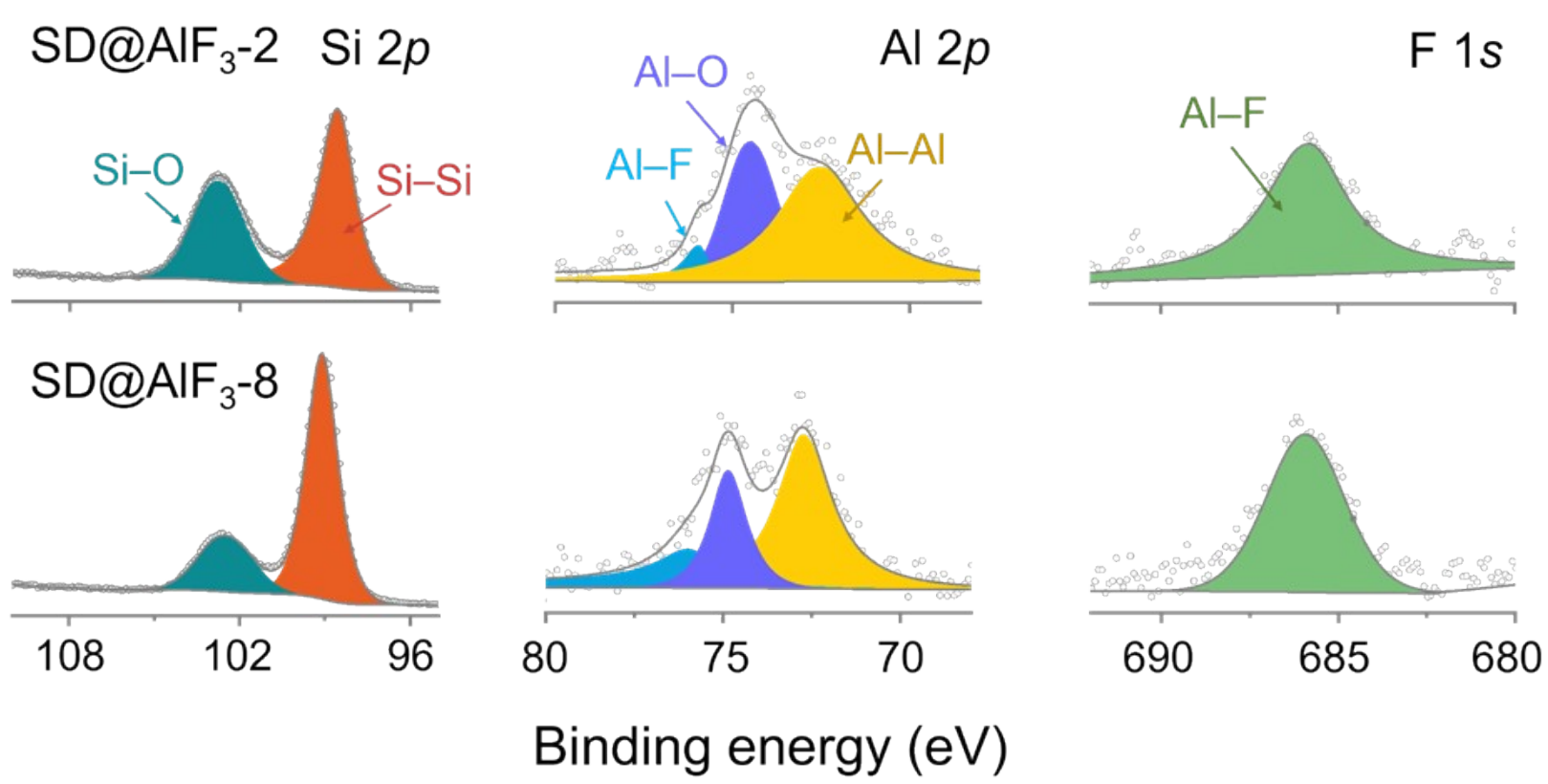

Fig. S7. High-resolution XPS spectra of SD@AlF_3-2 and SD@AlF_3-8, showing the Si 2p, Al 2p, and F 1s regions.

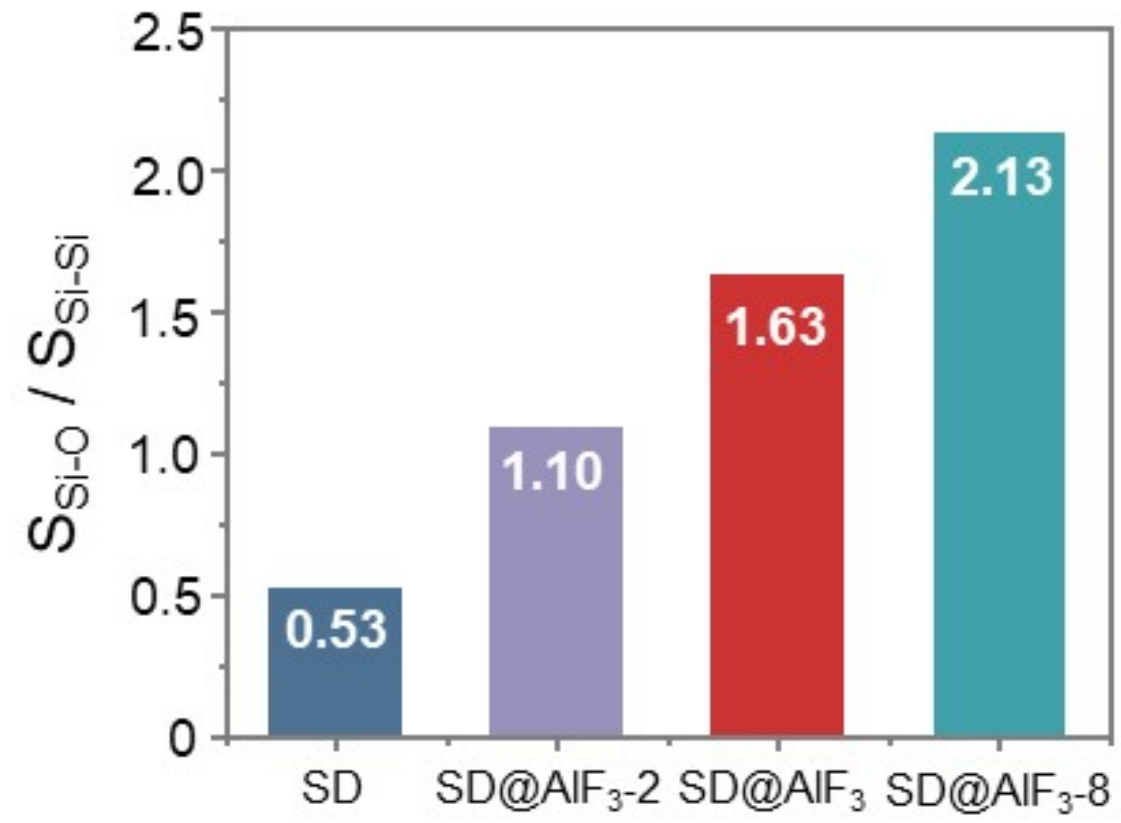

Fig. S8. Comparison of the peak area ratio of Si-O to Si-Si for SD, SD@AlF_3-2, SD@AlF_3, and SD@AlF_3-8.

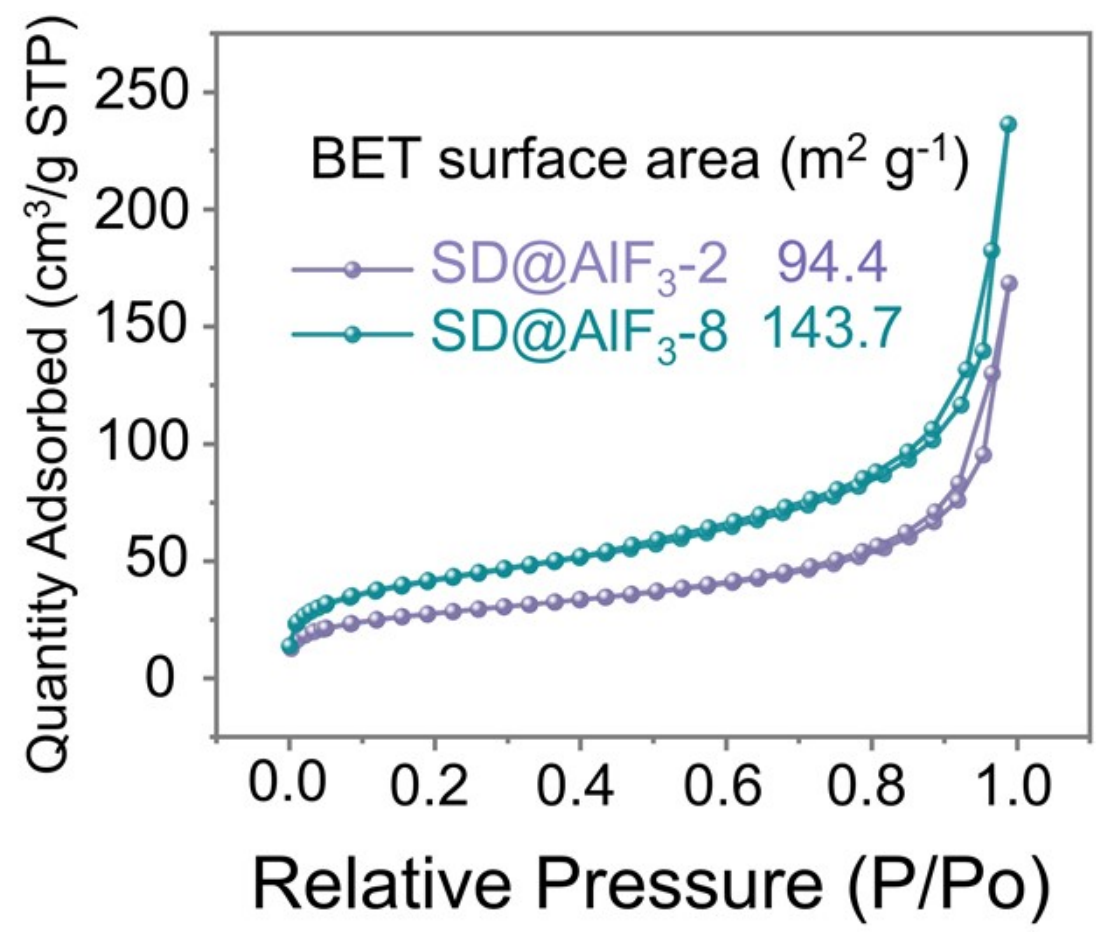

Fig. S9. N_2 adsorption-desorption isotherms of SD@AlF_3-2 and SD@AlF_3-8.

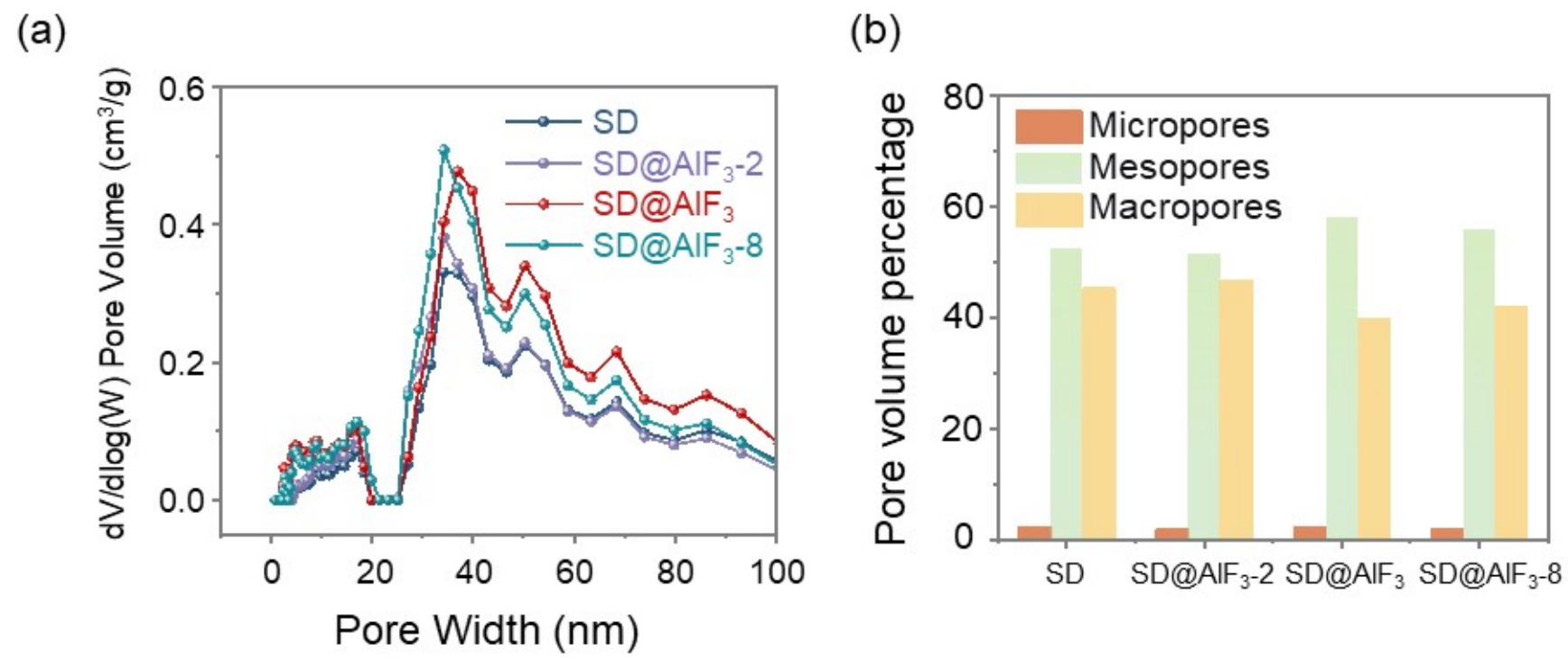

Fig. S10. (a) Pore size distribution curves (dV/dlogW) and (b) statistical histogram of pore volume fractions (micropores, mesopores, and macropores) for SD, SD@AlF_3-2, SD@AlF_3, and SD@AlF_3-8.

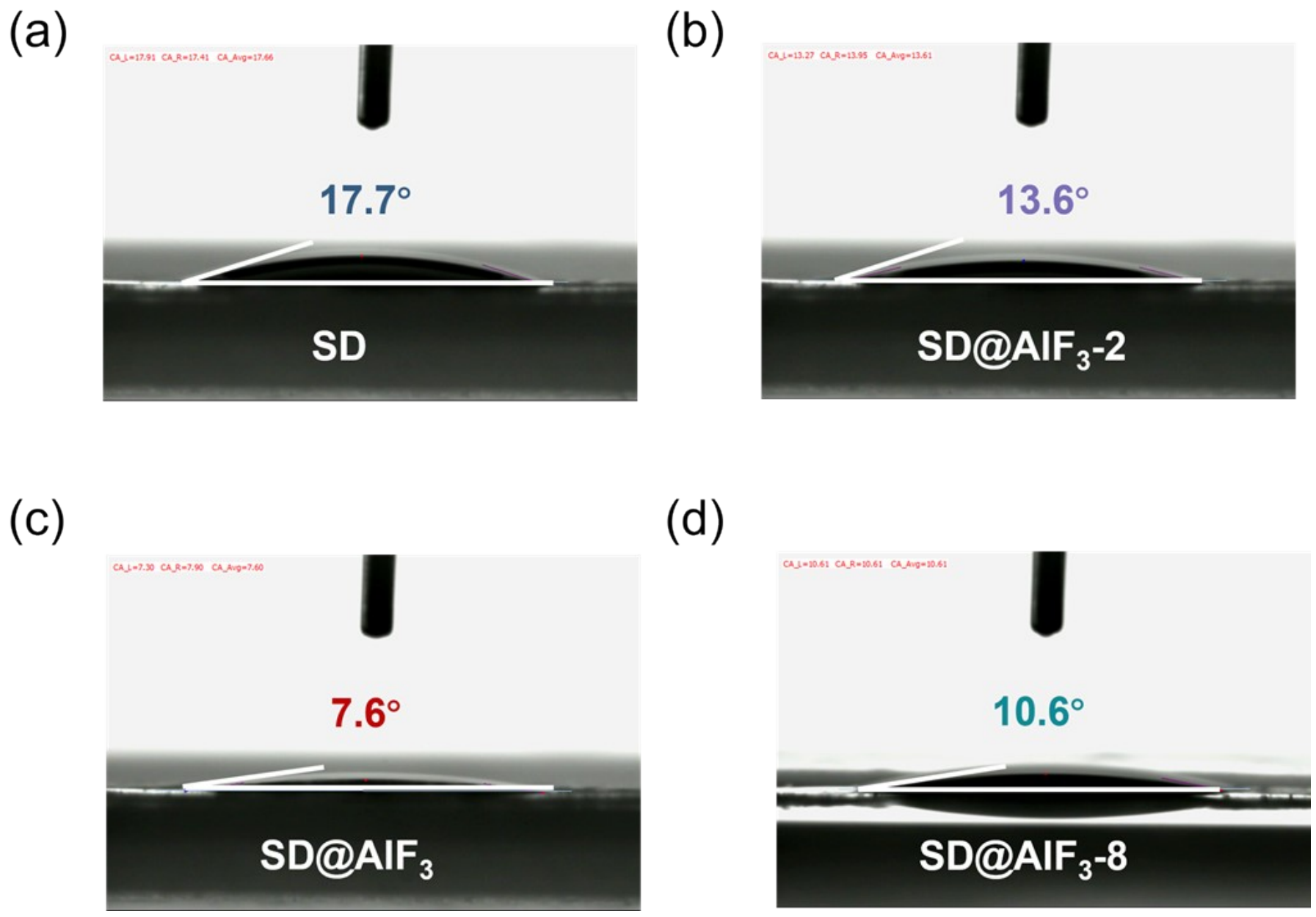

Fig. S11. Contact angle measurements using carbonate-based electrolyte (1 M $LiPF_6$ in EC/DEC (1:1 by volume) with 10 wt% FEC additive) for SD, SD@AlF_3-2, SD@AlF_3, and SD@AlF_3-8.

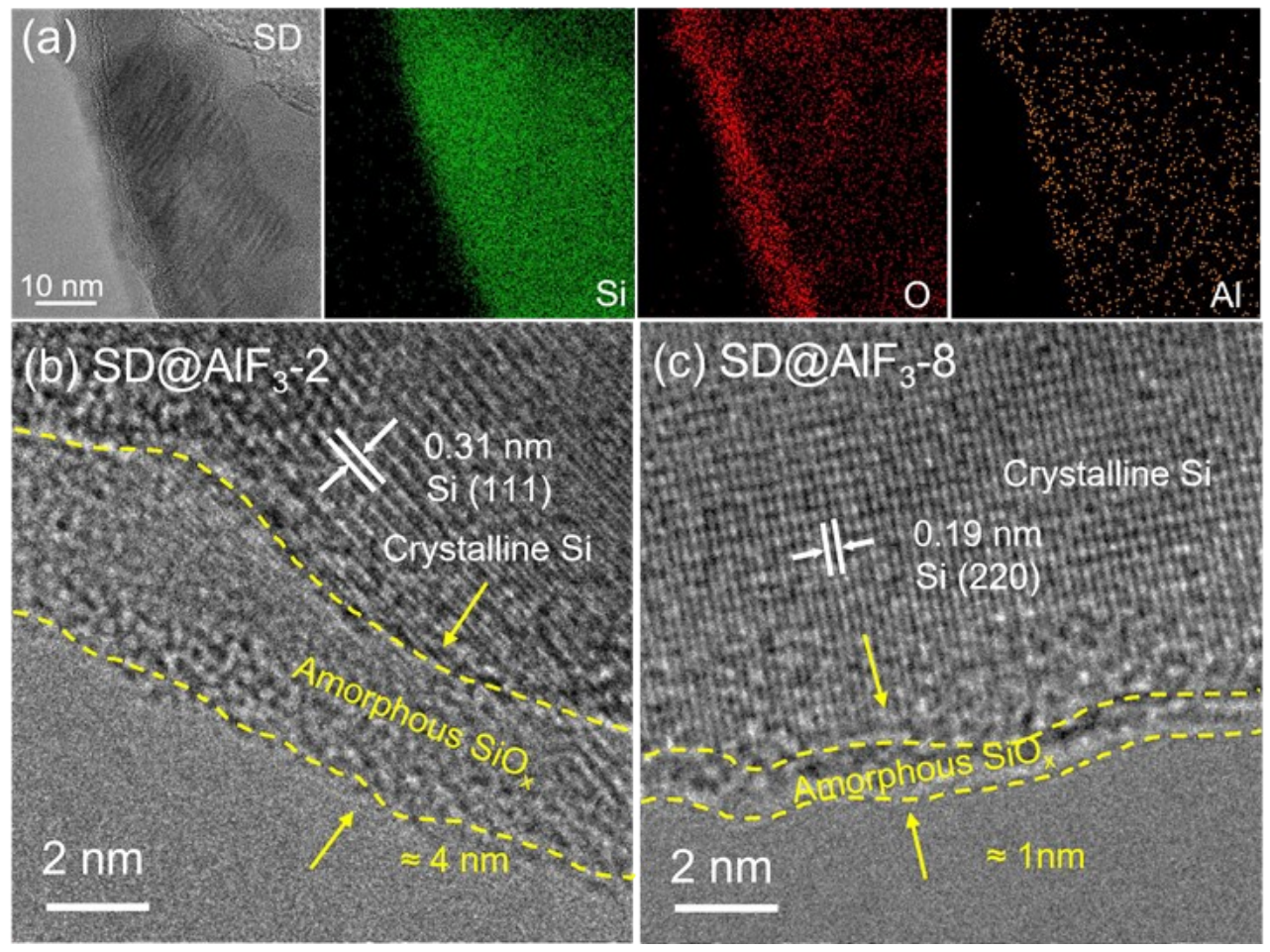

Fig. S12. (a) EDS elemental mapping of SD; HRTEM images of (b) SD@AlF_3-2 and (c) SD@AlF_3-8.

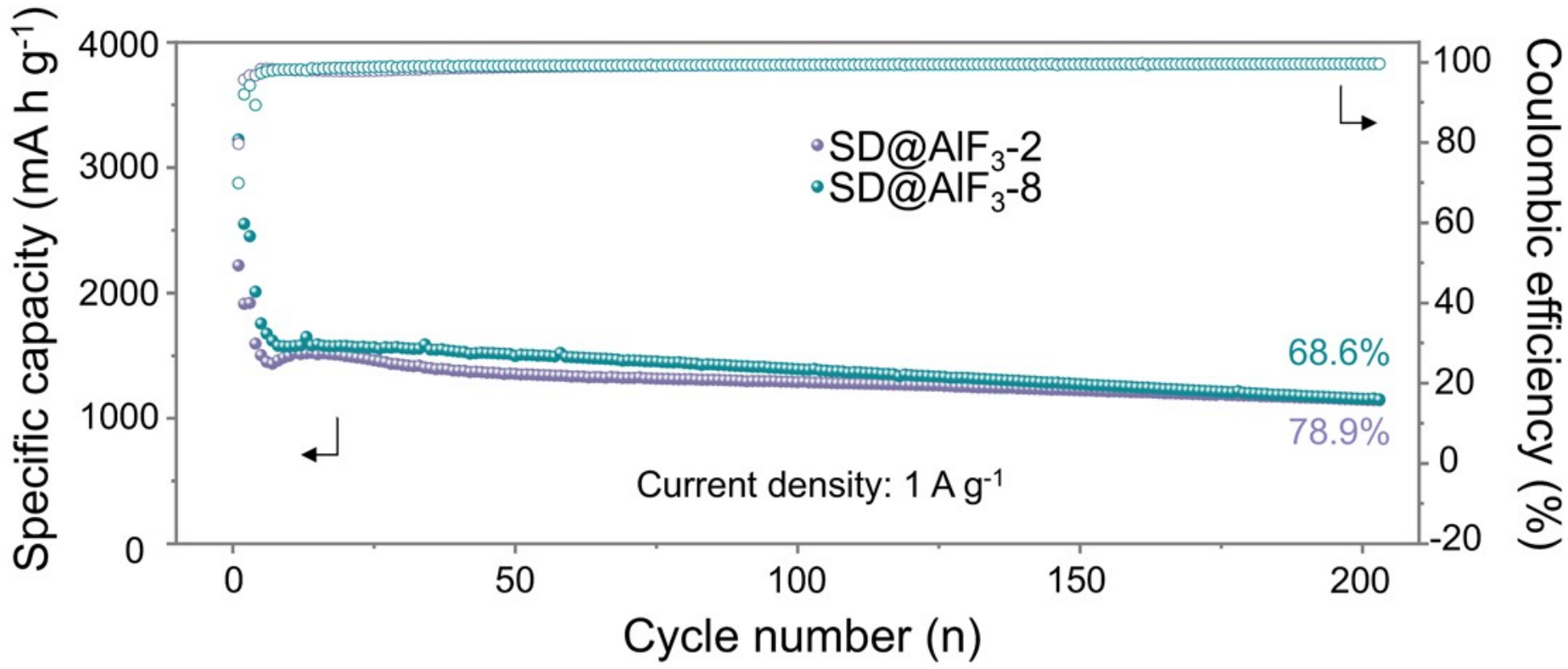

Fig. S13. Long-term cycling performance of SD@AlF_3-2 and SD@AlF_3-8 electrodes at a current density of 1 A g^{-1}.

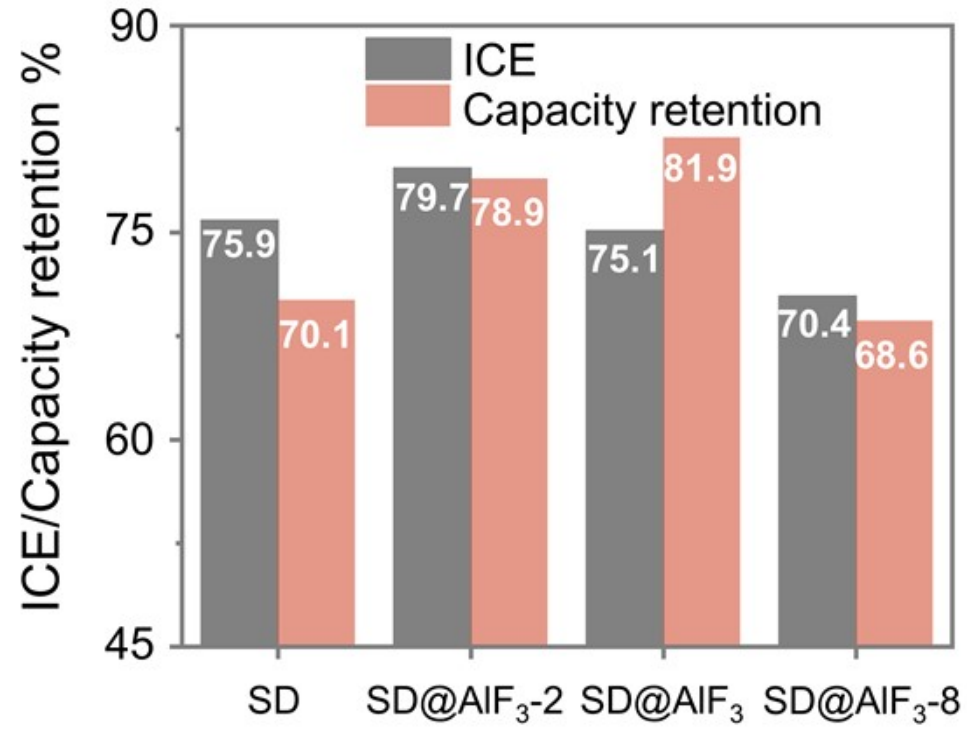

Fig. S14. Comparative histograms of (a) initial Coulombic efficiency (ICE) and (b) capacity retention after 200 cycles for SD, SD@AlF_3-2, SD@AlF_3, and SD@AlF_3-8.

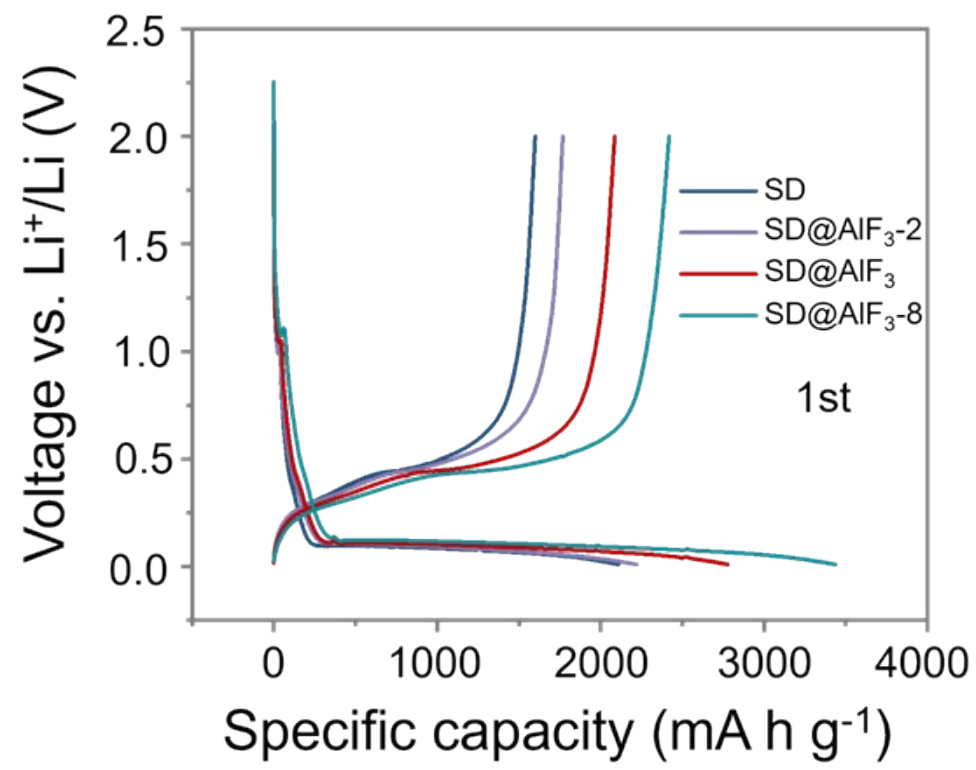

Fig. S15. Initial galvanostatic charge-discharge profiles of SD, SD@AlF_3-2, SD@AlF_3 and SD@AlF_3-8 at a current density of 0.2 A g^{-1}.

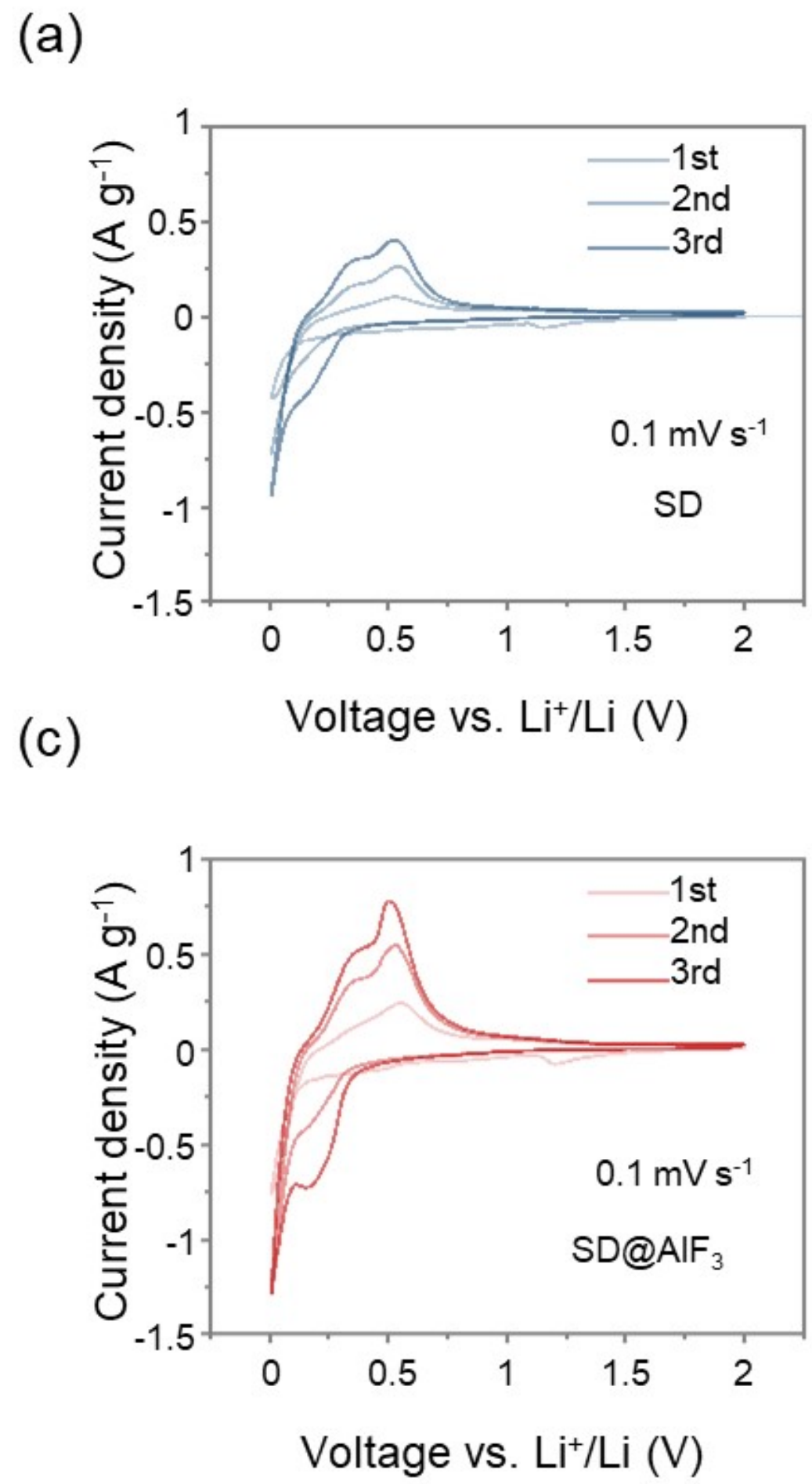

Fig. S16. Cyclic voltammetry (CV) curves of SD, SD@AlF_3-2, SD@AlF_3, and SD@AlF_3-8 for the first three cycles at a scan rate of 0.1 mV s^{-1}.

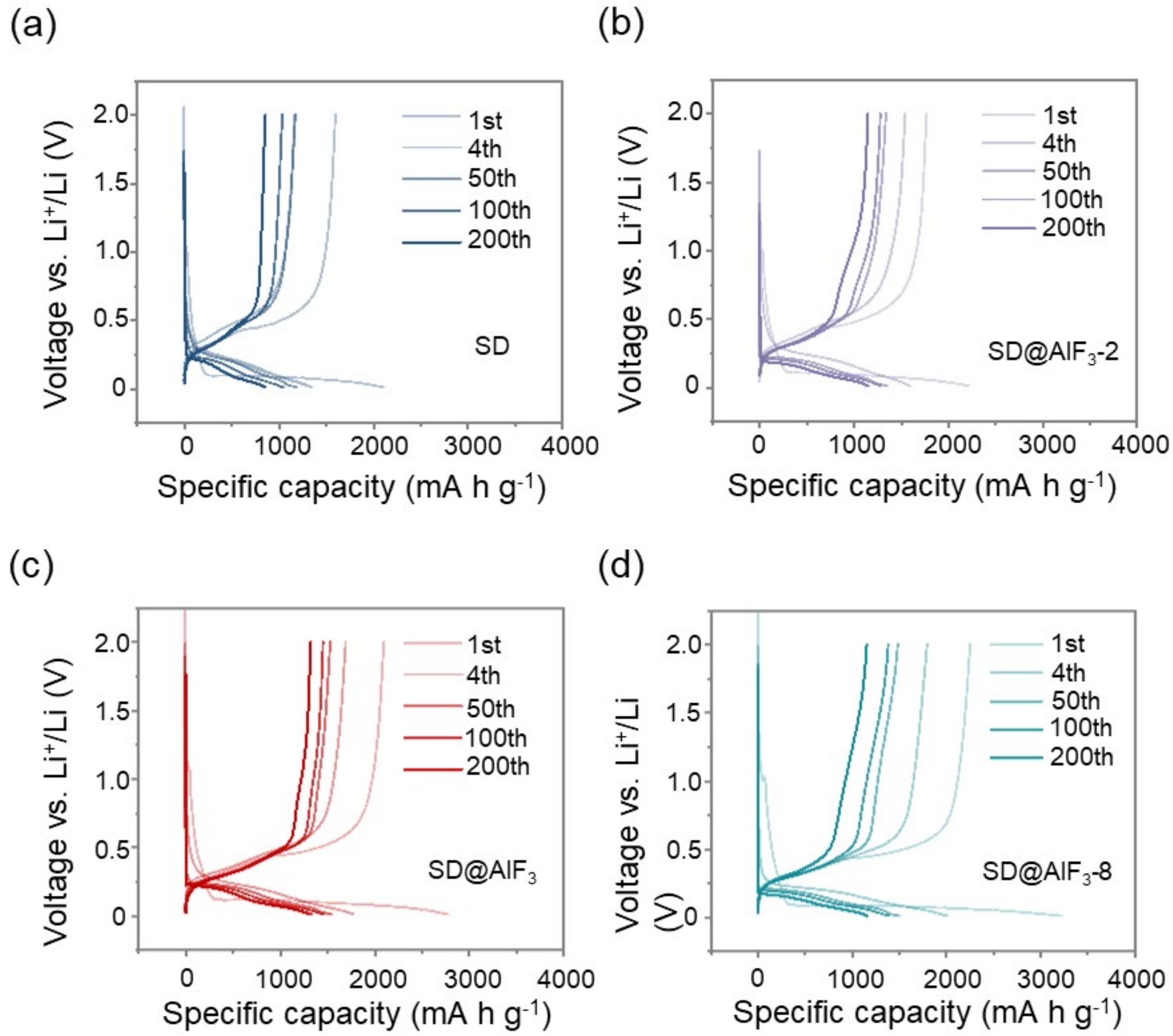

Fig. S17. Galvanostatic charge-discharge curves for the 1st, 4th, 50th, 100th, and 200th cycles of (a) SD, (b) SD@AlF_3-2, (c) SD@AlF_3, and (d) SD@AlF_3-8 electrodes.

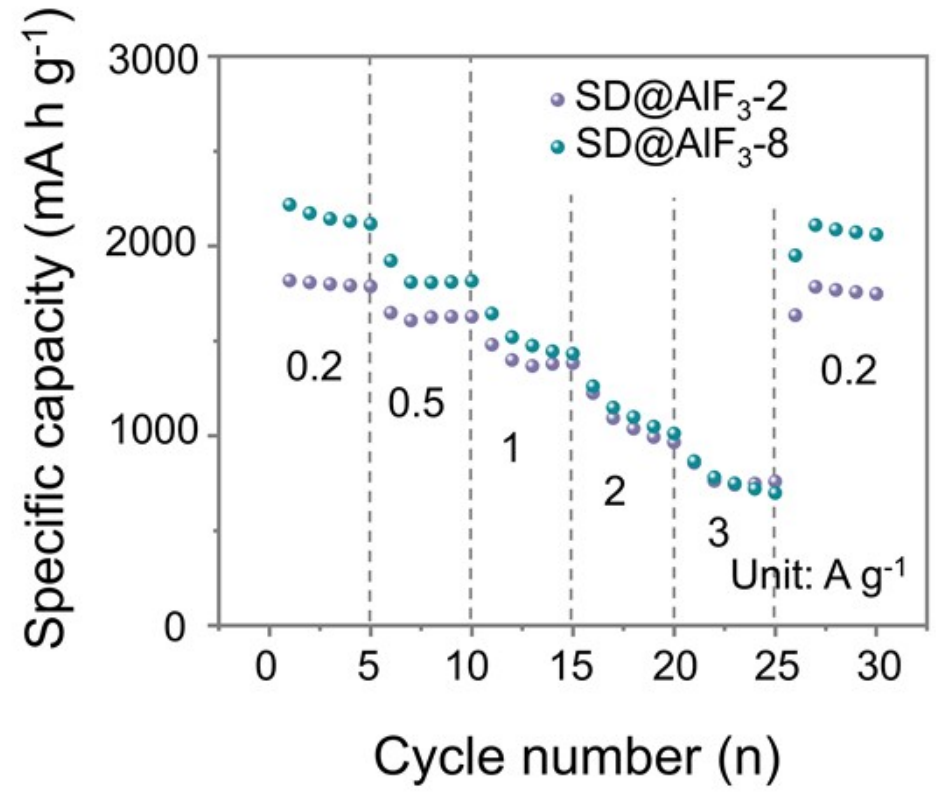

Fig. S18. Rate capability comparison of SD@AlF_3-2 and SD@AlF_3-8 at current densities ranging from 0.2 to 3 A g^{-1}.

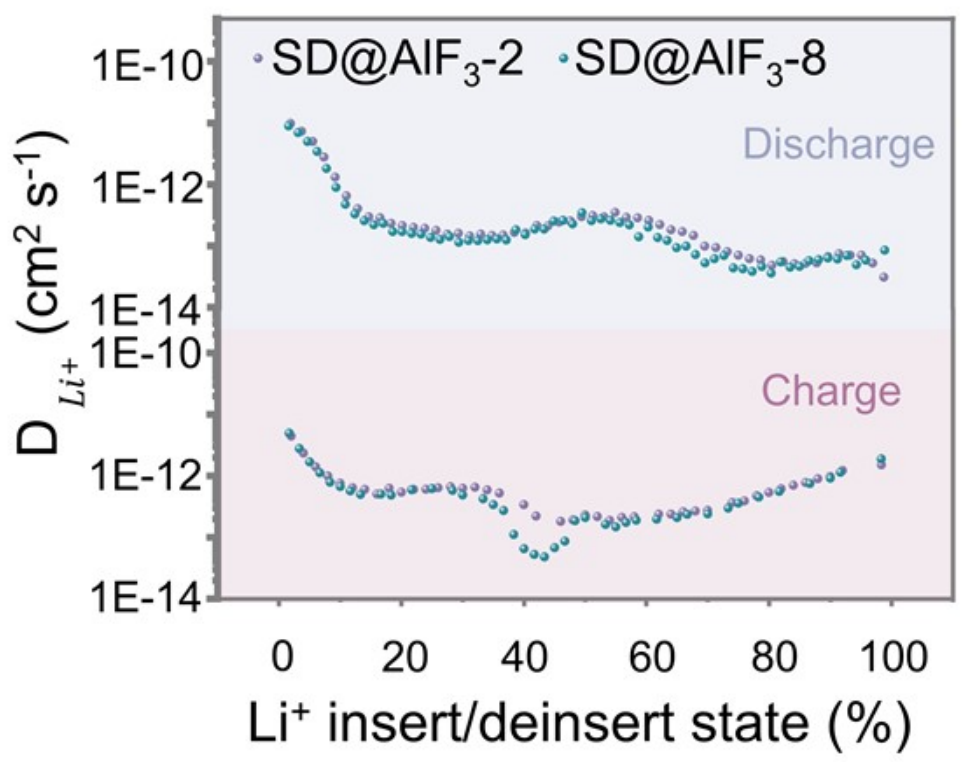

Fig. S19. GITT curves and calculated Li^+ diffusion coefficients for SD@AlF_3-2 and SD@AlF_3-8.

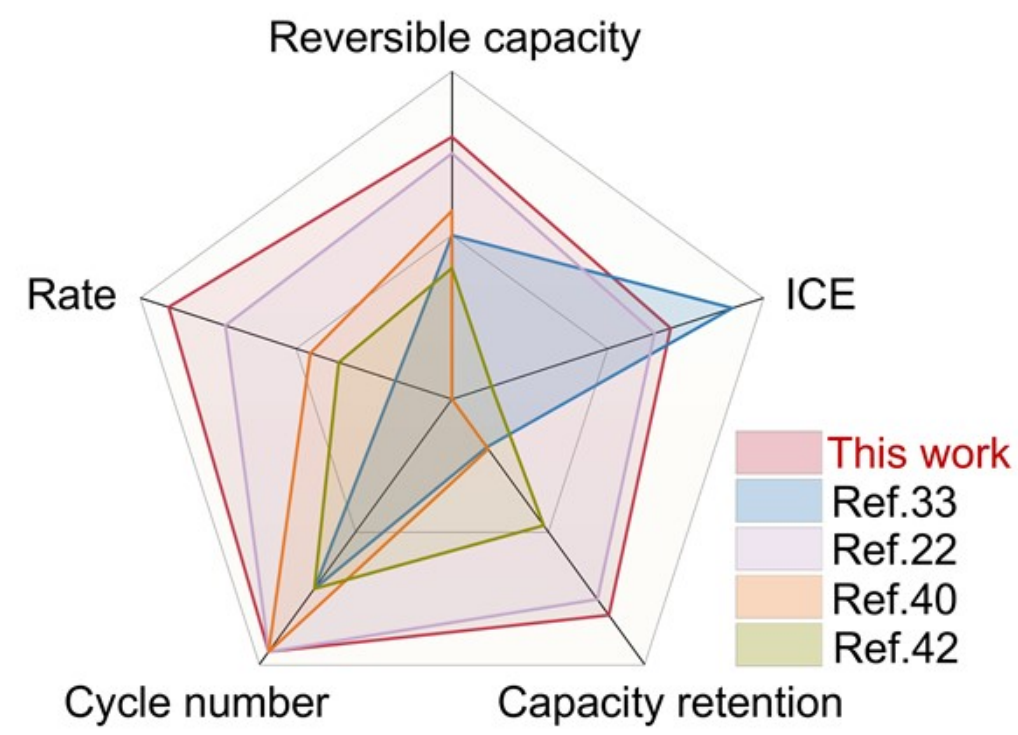

Fig. S20. Radar plot comparing the overall electrochemical performance of the SD@AlF_3 electrode with recently reported HF-etched or modified Si anodes.

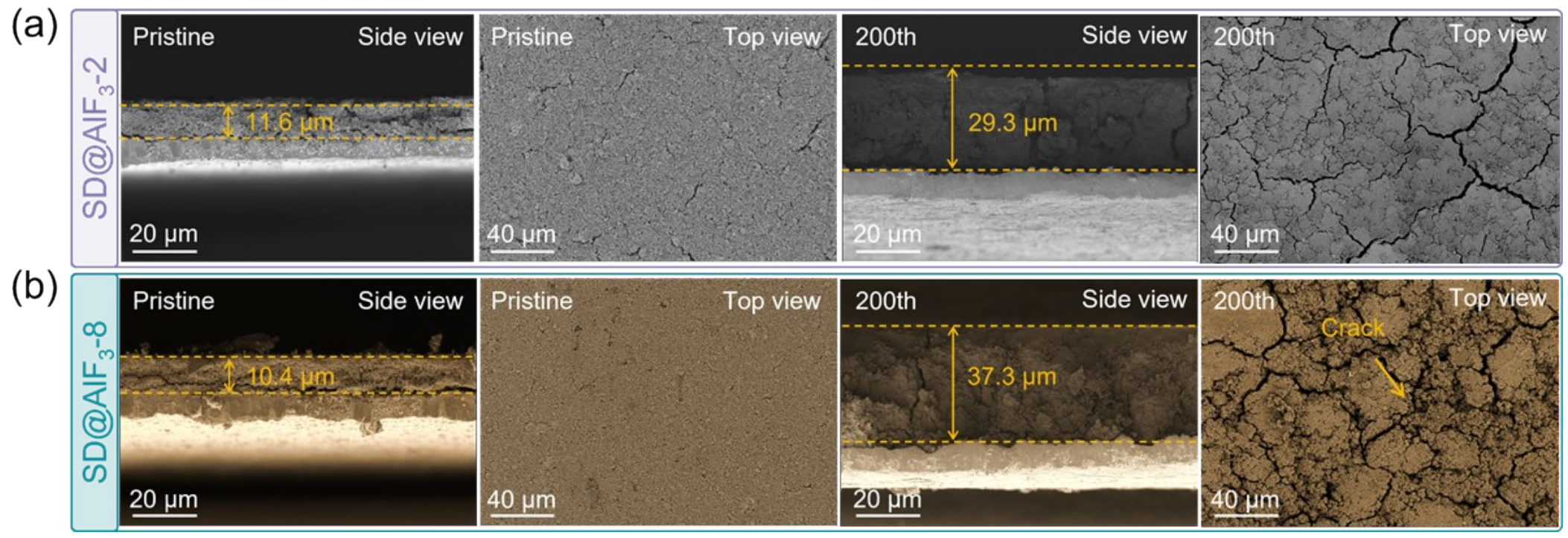

Fig. S21. Post-cycling SEM images (top-view and side-view) of (a) SD@AlF_3-2 and (b) SD@AlF_3-8 electrodes after 200 cycles.

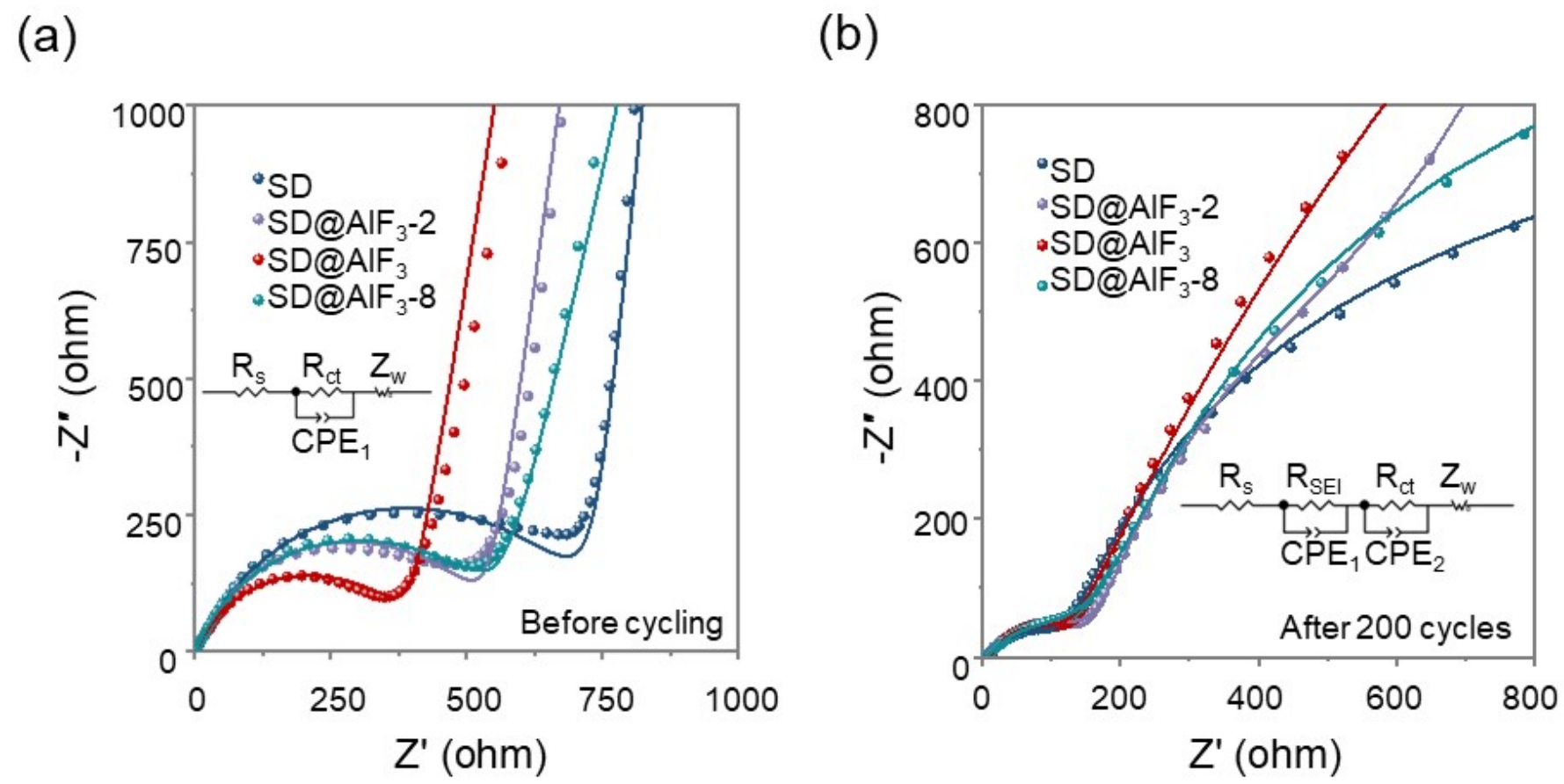

Fig. S22. Nyquist plots of SD, SD@AlF_3-2, SD@AlF_3, and SD@AlF_3-8 electrodes (a) before cycling and (b) after 200 cycles at 1 A g^{-1}.

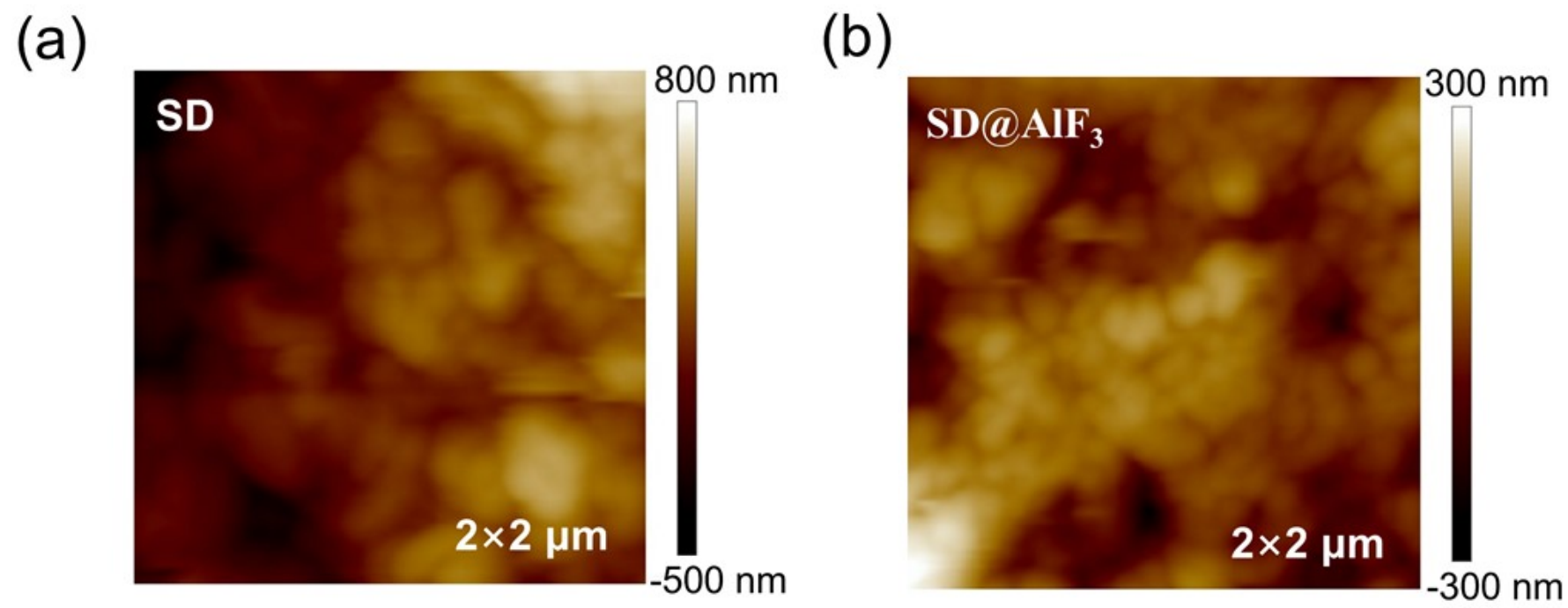

Fig. S23. AFM topographic images and surface roughness (R_a) analysis of cycled SD and SD@AlF_3.

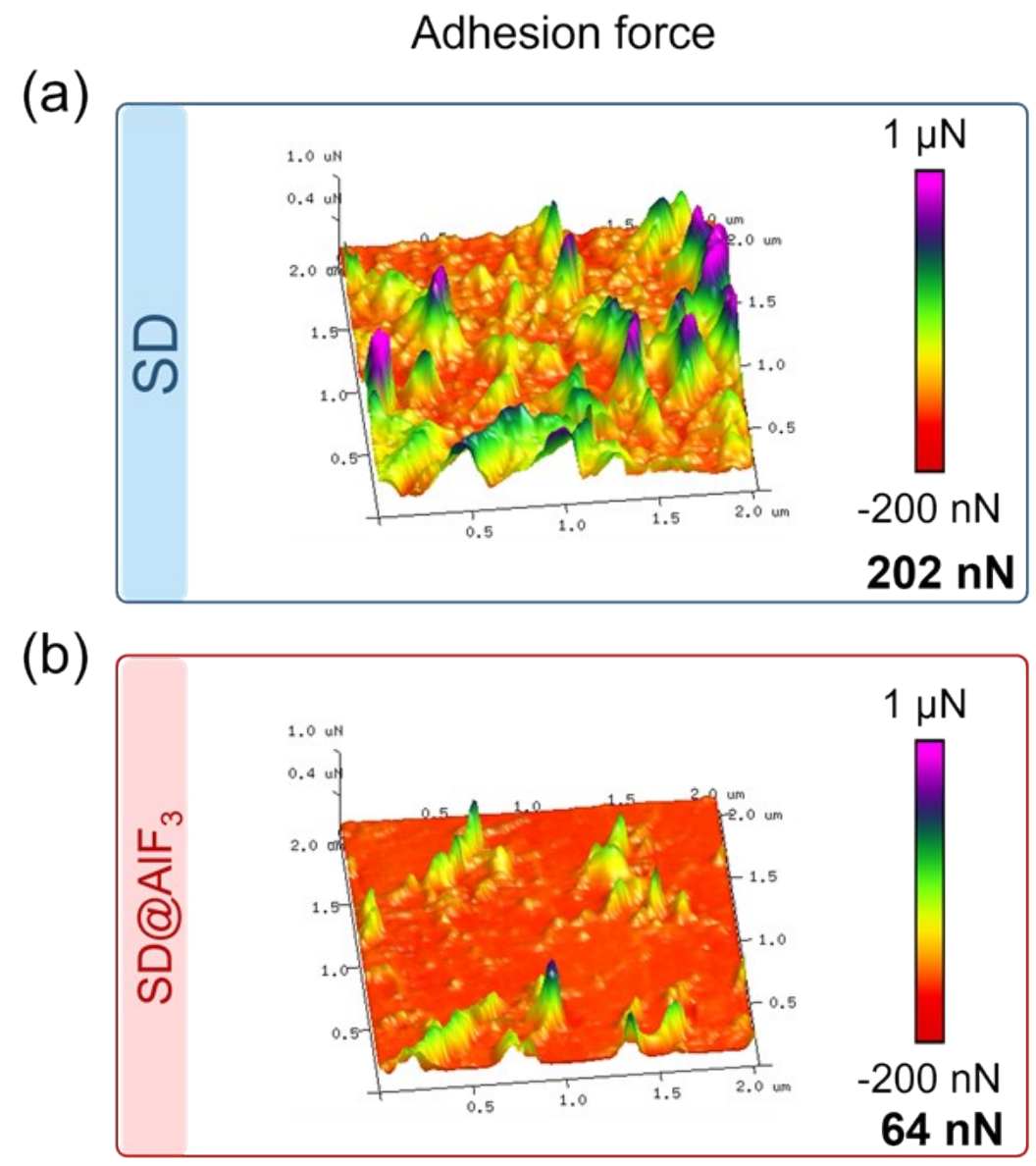

Fig. S24. AFM adhesion force maps of the SEI on cycled (a) SD and (b) SD@AlF_3 electrodes.

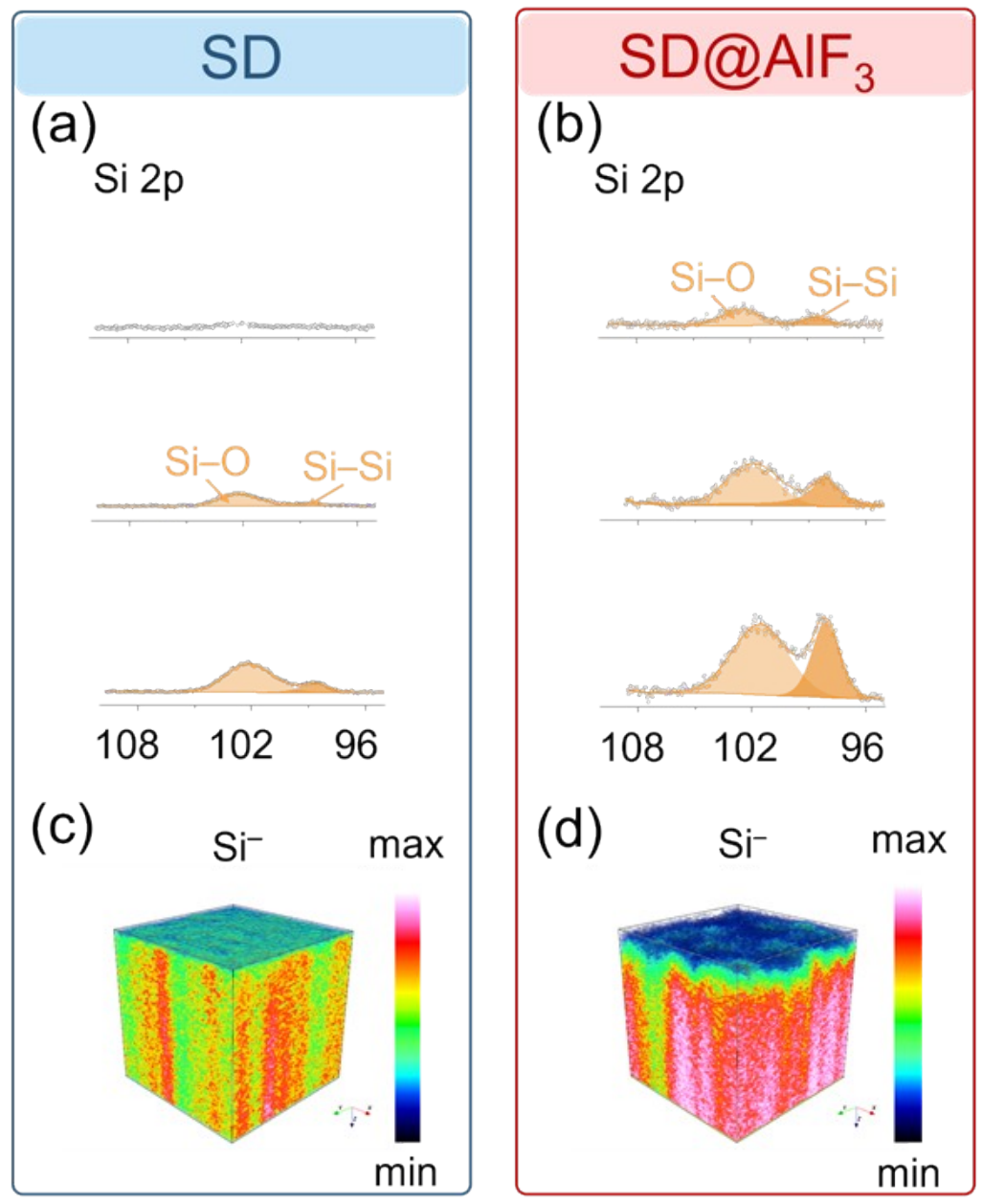

Fig. S25. (a-b) Depth-profiled Si 2p XPS spectra and (c-d) TOF-SIMS 3D ion maps of Si^- species for cycled SD and SD@AlF_3.

Table S1. Elemental composition from EDS analysis of SD, SD@AlF_3-2, SD@AlF_3, and SD@AlF_3-8.

Element (wt%)	SD	SD@AlF_3-2	SD@AlF_3	SD@AlF_3-8
O	43.2	33.3	27.9	22.0
F	0	2.1	3.5	3.7
Al	5.1	5.0	5.1	4.9
Si	51.7	59.6	63.5	69.4

Table S2. Textural parameters from N_2 physisorption for SD, SD@AlF_3-2, SD@AlF_3, and SD@AlF_3-8: pore volume distribution by pore size.

Pore Size Regime	SD	SD@AlF_3-2	SD@AlF_3	SD@AlF_3-8
Micropores (< 2 nm)	2.4%	1.9%	2.3%	2.1%
Mesopores (2-50 nm)	52.3%	51.5%	58.0%	55.9%
Macropores (50-100 nm)	45.3%	46.6%	39.7%	42.0%

Table S3. Performance comparison of SD@AlF_3 with reported HF-etched Si anodes

Rate (A g^{-1})	ICE (%)	Reversible capacity (mA h g^{-1})	Cycle number	Capacity retention (%)	Ref.
0.8	72.5	1500	200	71.7	22
0.2	85.0	1000	150	47.6	33
0.5	77.49	1950	132	80	38
0.1	/	1450	50	77.0	39
0.5	/	1150	200	47.9	40
0.5	74	1200	200	57.1	41
0.4	46.35	800	150	60	42
1.0	75.1	1600	200	81.9	This work

Table S4. The EIS fitting results of SD, SD@AlF_3-2, SD@AlF_3, and SD@AlF_3-8 before cycling and after 200 cycles.

Materials	Cycle	R_{ct} (Ω)	R_{SEI} (Ω)
SD	Before	697.6	/
SD@AlF_3-2	Before	503.6	/
SD@AlF_3	Before	394.4	/
SD@AlF_3-8	Before	560.3	/
SD	200th	1615.6	124.5
SD@AlF_3-2	200th	960.7	174.4
SD@AlF_3	200th	617.4	114.1
SD@AlF_3-8	200th	1916.5	158.3
